# *Cellulose synthase-like D1* controls organ size in maize

**DOI:** 10.1186/s12870-018-1453-8

**Published:** 2018-10-16

**Authors:** Weiya Li, Zhixing Yang, Jieyuan Yao, Jiansheng Li, Weibin Song, Xiaohong Yang

**Affiliations:** 1 State Key Laboratory of Plant Physiology and Biochemistry, China Agricultural University, Beijing, 100193 China; 2 National Maize Improvement Center of China, MOA Key Lab of Maize Biology, Beijing Key Laboratory of Crop Genetic Improvement, China Agricultural University, Beijing, 100193 China

**Keywords:** QTL, *ZmCSLD1*, Organ size, Pleiotropic effects and intragenic complementation

## Abstract

**Background:**

Plant architecture is a critical factor that affects planting density and, consequently, grain yield in maize. The genes or loci that determine organ size are the key regulators of plant architecture. Thus, understanding the genetic and molecular mechanisms of organ size will inform the use of a molecular manipulation approach to improve maize plant architecture and grain yield.

**Results:**

A total of 18 unique quantitative trait loci (QTLs) were identified for 11 agronomic traits in the F_2_ and F_2:3_ segregating populations derived from a cross between a double haploid line with a small plant architecture (MT03-1) and an inbred line with a large plant architecture (LEE-12). Subsequently, we showed that one QTL, *qLW10*, for multiple agronomic traits that relate to plant organ size reflects allelic variation in *ZmCSLD1*, which encodes a cellulose synthase-like D protein. ZmCSLD1 was localized to the *trans*-Golgi and was highly expressed in the rapidly growing regions. The loss of *ZmCSLD1* function decreased cell division, which resulted in smaller organs with fewer cell numbers and, in turn, pleiotropic effects on multiple agronomic traits. In addition, intragenic complementation was investigated for two *Zmcsld1* alleles with nonsynonymous SNPs in different functional domains, and the mechanism of this complementation was determined to be through homodimeric interactions.

**Conclusions:**

Through positional cloning by using two populations and allelism tests, *qLW10* for organ size was resolved to be a cellulose synthase-like D family gene, *ZmCSLD1*. *ZmCSLD1* has pleiotropic effects on multiple agronomic traits that alter plant organ size by changing the process of cell division. These findings provide new insight into the regulatory mechanism that underlies plant organ development.

**Electronic supplementary material:**

The online version of this article (10.1186/s12870-018-1453-8) contains supplementary material, which is available to authorized users.

## Background

Maize (*Zea mays* ssp. *mays*) is the highest yielding and most widely grown crop in the world. Increasing grain yield has always been a primary goal of maize breeding. Plant architecture is a result of many trait interactions during plant development and growth, and is a critical factor that affects plant density and, consequently, grain yield. In general, maize plant architecture includes plant height (PH), leaf number (LN), leaf angle (LA), leaf area, and tassel traits. An ideal plant architecture includes a short stature to prevent lodging, an erect LA and a moderate leaf area to maximize light interception, and small tassels to partition more energy and nutrients for ear and seed development [1–4]. Most of the traits that contribute to plant architecture, such as PH, leaf length (LL), leaf width (LW), and tassel length (TL) can collectively be referred to as organ size, which is determined by cell number and cell size and results from the two successive processes of cell proliferation and cell expansion. Thus, understanding the genetic and molecular mechanism of organ size will inform the development of a molecular manipulation approach to improve maize plant architecture and grain yield.

Plant organ size is a product of the interaction of genotype and environmental influences [5]. The genetic architecture of organ size has been well studied in maize by using different genetic populations [6–11]. For example, a nested association mapping (NAM) population, which contained ~ 5,000 recombinant inbred lines and was developed by crossing 25 diverse lines to B73, was used to identify the quantitative trait loci (QTLs) of multiple traits that relate to organ size [7, 9, 10]. By using joint linkage analysis, a total of 89, 92, 37, 36, and 34 QTLs were detected for PH, ear height (EH), TL, LL and LW, respectively. Plant architecture traits were also studied in a natural population that contained 513 diverse maize lines by a genome-wide association study, and 185 SNPs were significantly associated with PH, EH, LL, LW and TL [11]. Recently, approximately 314 QTLs were identified to control the phenotypic variation in organ size-related traits (PH, EH, LL and LW) in 10 maize populations [8]. These QTL studies indicate that the formation of organ size is complex and characterized by small effects for most QTLs, large effects for several QTLs, and a certain number of pleiotropic QTLs.

Studies of the model plant *Arabidopsis* have revealed that the formation of plant organ size are modulated by several molecular mechanisms, including transcriptional regulation [12–15], hormone signalling [15–18], ubiquitin-mediated proteolysis [19, 20], and cell wall biosynthesis [21]. However, only several genes for organ size have been isolated and characterized in maize, such as *BR2* [22, 23] and *ZmGA3ox2* or *D1* [24, 25] for PH; *DIL1*, *GA20-OXIDASE1*, *GRF1* and *GRF10* for LL and PH [26–29]; *ZmGE2* [30] and *BIGE1* [31] for embryo size; and *ZmBRI1*, *CNR1*, *CNR2* and *ZmPLA1* for overall organ size [32–34]. Similar to *Arabidopsis*, organ size in maize is controlled by multiple genes in various regulatory pathways. For the genes known to regulate organ size, the molecular mechanisms of most genes regulating organ size relate to hormone signalling [23, 25, 34]. As expected, all the genes control organ size by changing cell division and cell expansion to alter cell number or cell size, respectively. For example, overexpressing *ZmPLA1* increased organ size by prolonging cell division, and knocking down *ZmBRI1* reduced organ size by decreasing cell division and cell elongation [32, 34]. Besides, the regulation ways to organ growth were also different in the same tissue. Both *BR2* and *ZmGA3ox2* control PH, although, *BR2* plays a role in cell division, while *ZmGA3ox2* plays a role in cell expansion [23, 25]. Similarly, LL was regulated by *GRFs*, with overexpression of *GRF1*^*R*^ and *GRF10* increasing LL by cell division and expansion, respectively [28, 29]. In addition, not like the role of *ZmGA3ox2* in stem, gibberellin greatly enlarged the cell division zone, and increased LL in *GA20-OXIDASE1* overexpression plants [27, 35]. These results had provided summary knowledge about the genes that control organ size, although the regulatory pathways in which these genes operate are still poorly understood in maize.

In this study, we developed a double haploid line with a small plant architecture, MT03-1, which was crossed with an inbred line with a large plant architecture, LEE-12, to determine the genetic architecture of the maize agronomic traits that relate to organ size. A major QTL for organ size, *qLW10*, was identified in the F_2_ and F_2:3_ segregating populations derived from a cross between MT03-1 and LEE-12. This locus was further cloned to be *ZmCSLD1*, which encodes a cellulose synthase-like D1 protein that affects plant growth through cell division or expansion. The molecular mechanism of ZmCSLD1 that affects plant organ size was extensively addressed through genetic analysis, subcellular localization, an expression profile and the analysis of cell number and cell size.

## Results

### A large-effect QTL cluster controls multiple agronomic traits

**Fig. 1:**
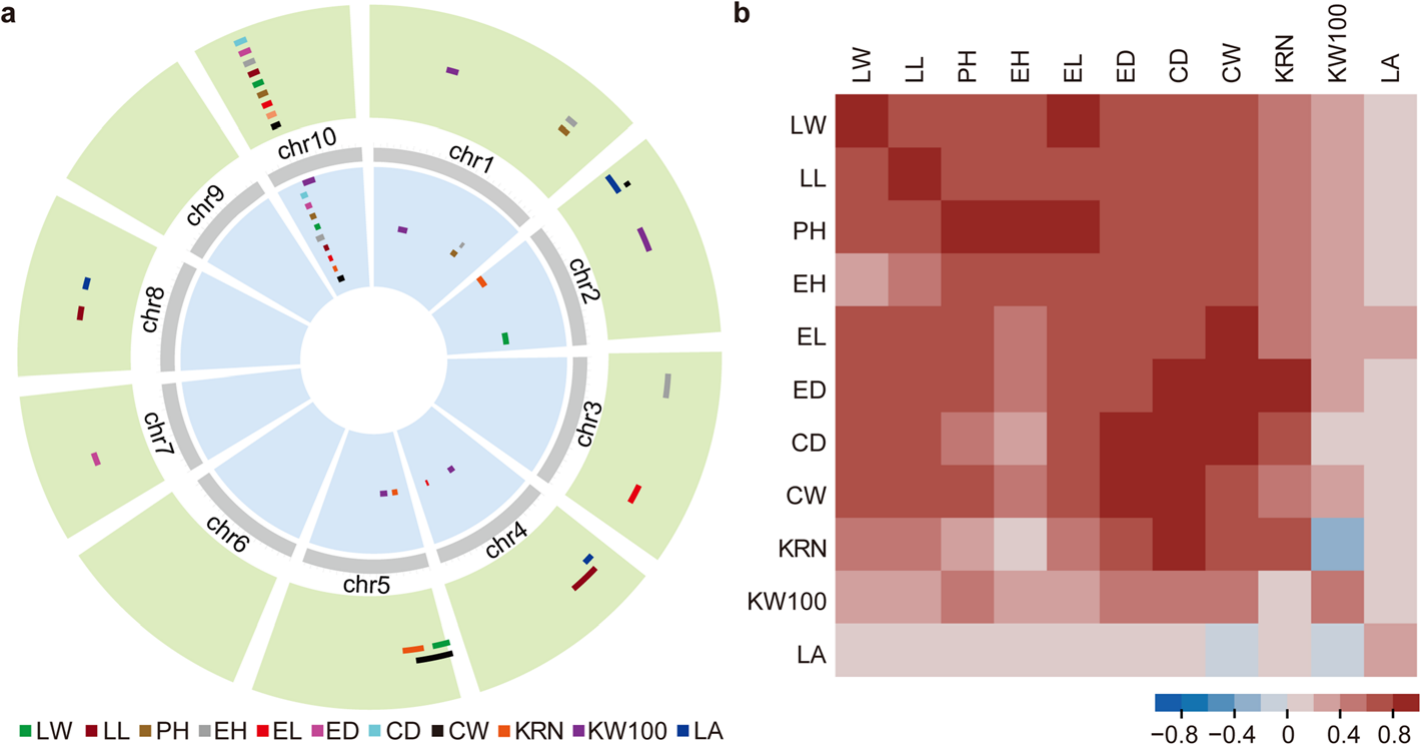
QTLs for 11 agronomic traits identified in the LEE-12 × MT03-1 F_2_ and F_2:3_ segregating populations. **a** Chromosomal distribution of QTLs for the 11 traits. The outermost circle represents the distribution of QTLs identified in the F_2_ population, and the innermost circle represents the distribution of QTLs identified in the F_2:3_ population. The circle beneath the scale represents the genetic position on the linkage map. **b** Pearson correlation among different traits. The lower left indicates the correlation coefficient in the F_2_ population, the upper right indicates the correlation coefficient in the F_2:3_ population, and the diagonal is the correlation coefficient of the same trait between F_2_ and F_2:3_. The abbreviations and detailed descriptions of the 11 traits are listed in Additional file 2: Table S2

MT03-1 is a double haploid line with a relatively small plant architecture (Additional file 1: Table S1). To investigate the genetic basis of these traits, QTL mapping was performed for the 11 measured traits by using a genetic map of 1833.66 cM (Additional file 1: Tables S1; Additional file 2: Tables S2), and a total of 19 and 25 QTLs were detected in the F_2_ and F_2:3_ populations, respectively (Fig. 1a; Additional file 3: Table S3). These QTLs were distributed in 18 genomic regions across 8 of the 10 chromosomes, except for chromosomes 6 and 9, and each QTL explained 3.27 to 75.05% of the phenotypic variation. Out of 18 unique QTLs, 6 and 2 were associated with at least two traits in the F_2_ and F_2:3_ populations, respectively, which indicates that they had pleiotropic effects or were closely linked loci.

Among these QTLs, one QTL cluster with a large effect on chromosome 10, which was flanked by SYN17100 and PZE110041758, was identified for nine traits in both the F_2_ and F_2:3_ populations. On average, this locus explained 27.35% of the phenotypic variation in all nine traits and differed from 75.05% for LW to 9.18% for EH. In addition, all the alleles at this locus associated with decreased size came from MT03-1. This result was highly consistent with the phenotypic correlation among traits (Fig. 1b). All nine traits with the QTL identified at this locus had a moderate to high correlation with one another (*r* = 0.44–0.94, F_2:3_ population), whereas the two remaining traits without the identified QTL at this locus, namely, the LA and 100-kernel weight (KW100), were weakly correlated with the nine traits (*r* = - 0.2–0.34, F_2:3_ population). This result suggests that the nine traits may share a common pleiotropic genetic variant at this locus.

### *ZmCSLD1* is the causal gene for *qLW10*

**Fig. 2:**
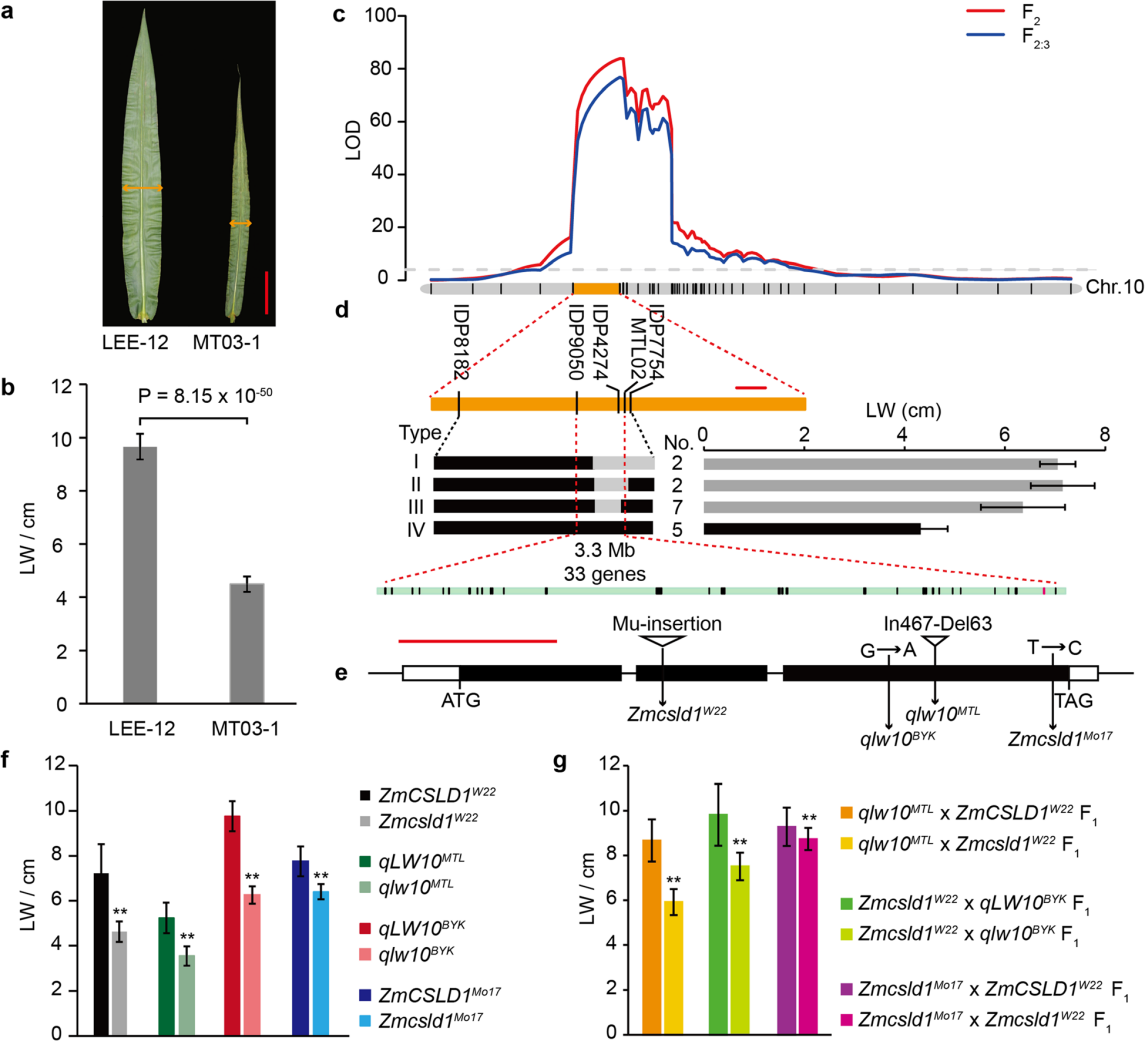
Positional cloning of *qLW10* by using LEE-12 × MT03-1 segregating populations. **a** Mature ear leaf of the parents MT03-1 and LEE-12. Scale bar = 10 cm. **b** Statistical analysis of the LW between MT03-1 and LEE-12. **c** LOD profile of *qLW10*, which was identified in the F_2_ and F_2:3_ populations. **d** *qLW10* was mapped to a 3.3-Mb genomic DNA region between the markers IDP9050 and MTL02 by using 3,292 BC_2_F_1_ and 669 BC_3_F_1_ plants. The numbers (Num) and LW are shown for the recombinant plants (I–III) and the non-recombinant plants (IV). The black and grey bars represent the chromosomal segments for the homozygous MT03-1 and heterozygous alleles, respectively. The data are shown as the mean ± SD. Scale bar = 2 Mb. **e** Four allelic variations in the candidate gene *ZmCSLD1*; the insertions (triangles) and point mutations are shown. Scale bar = 1 kb. **f**–**g** LW in the lines with four pairs of allelic samples (**f**) and three pairs of F_1_ hybrids (**g**). The data are shown as the mean ± SD (*n* = 30). **P < 0.01 (Student’s t-test)

To clone the causal gene for the QTL cluster, we selected LW as the trait for positional cloning because the greatest difference was observed for LW between the parents, LEE-12 and MT03-1 (Fig. 2a and b; Additional file 1: Table S1), and this locus had the largest effect on LW (Fig. 2c; Additional file 3: Table S3). This locus was then designated *qLW10*. The position of *qLW10* was refined to a 3.3-Mb segment flanked by markers IDP9050 and MTL02 by using 3,292 BC_2_F_1_ and 669 BC_3_F_1_ plants (Fig. 2d). To confirm the narrowed interval, a heterozygous inbred family BYK-HIF, which segregated in LW (Additional file 4: Figure S1A-C) was also used to fine map *qLW10*. Nine recombinant types were identified in 720 F_2_ plants selfed from BYK-HIF, and the progeny test of homozygous segregants narrowed the interval to a 3.8-Mb region flanked by the markers IDP9050 and IDP7754 (Additional file 4: Figure S1D). The nucleotide sequence analysis based on B73 reference sequences predicted 33 and 36 genes in the 3.3- and 3.8-Mb intervals, respectively. Simultaneously, two pairs of near-isogenic lines (NILs) were developed. *qLW10*^*MTL*^ and *qlw10*^*MTL*^ carried 3.3-Mb fragments from LEE-12 and MT03-1, respectively. *qLW10*^*BYK*^ and *qlw10*^*BYK*^ harboured different alleles at the target locus, with only a 0.4% difference in the genome background. Compared with *qLW10*^*MTL*^ and *qLW10*^*BYK*^, *qlw10*^*MTL*^ and *qlw10*^*BYK*^ had smaller plant architectures (Additional file 5: Figure S2; Additional file 6: Figure S3).

Among these genes in the *qLW10* interval, GRMZM2G015886 (*ZmCSLD1*) has been previously reported to narrow LW and reduce growth after a loss of function [36], which is very similar to the effect of *qLW10*. Therefore, *ZmCSLD1* was proposed as the best candidate gene for *qLW10*. To confirm this hypothesis, we first sequenced the full-length *ZmCSLD1* in two pairs of NILs. Compared with the *qLW10*^*MTL*^ allele, the *qlw10*^*MTL*^ allele contained a 467-bp insertion and a 63-bp deletion in the third exon, resulting in a frameshift that prevented the production of the mature protein because of the introduction of a premature stop codon (Fig. 2e). For the *qLW10*^*BYK*^ and *qlw10*^*BYK*^ lines, there was a G to A transition in the third exon, causing a conserved amino acid with a major change from glycine to aspartic acid (Fig. 2e; Additional file 4: Figure S1E; Additional file 7: Figure S4).

To confirm that *ZmCSLD1* is the causal gene for *qLW10*, two additional *Zmcsld1* mutants, *Zmcsld1*^*MO17*^ and *Zmcsld1*^*W22*^, were developed (Fig. 2e). A genomic sequence analysis revealed that *Zmcsld1*^*MO17*^ had a T-to-C substitution (tryptophan-to-arginine) in the third exon and a conserved amino mutation in the last transmembrane domain of ZmCSLD1 (Additional file 7: Figure S4), and *Zmcsld1*^*W22*^ had a Mu-transposon insertion in the second exon. As expected, the LW in each mutant was significantly decreased compared with their corresponding wild-type lines, similar to *qlw10*^*MTL*^ and *qlw10*^*BYK*^ (Fig. 2f). The allelic effect was the largest for *Zmcsld1*^*W22*^, followed by *qlw10*^*MTL*^, *qlw10*^*BYK*^ and *Zmcsld1*^*Mo17*^. To further evaluate the allelic effect, *Zmcsld1*^*W22*^ was crossed with *qlw10*^*MTL*^, *qlw10*^*BYK*^, *Zmcsld1*^*MO17*^ and their wild types, as designated in Additional file 8: Table S4. Compared with the F_1_ hybrids with a normal allele, all three F_1_ hybrids with both mutant alleles had narrower leaves (Fig. 2g), which was further validated in the six F_2_ populations produced by the four mutant combinations (Additional file 9: Figure S5A and C). These results indicate that *ZmCSLD1* is the causal gene for *qLW10*.

### Characterization of *ZmCSLD1*

ZmCSLD1 is a member of the conserved family of CSLD proteins in maize that are important for cell growth and development [36]. ZmCSLD1 contains a “D,D,D,QXXRW” motif with a RING-type zinc finger-like domain and two transmembrane domains at the N-terminus and six transmembrane domains in the C-terminal region (Additional file 7: Figure S4). The phylogenetic tree of *ZmCSLD1* orthologues across 32 angiosperms indicate that ZmCSLD1 is highly conserved in different classes such as asterids, core eudicots, rosids and poales (Additional file 10: Figure S6).

**Fig. 3:**
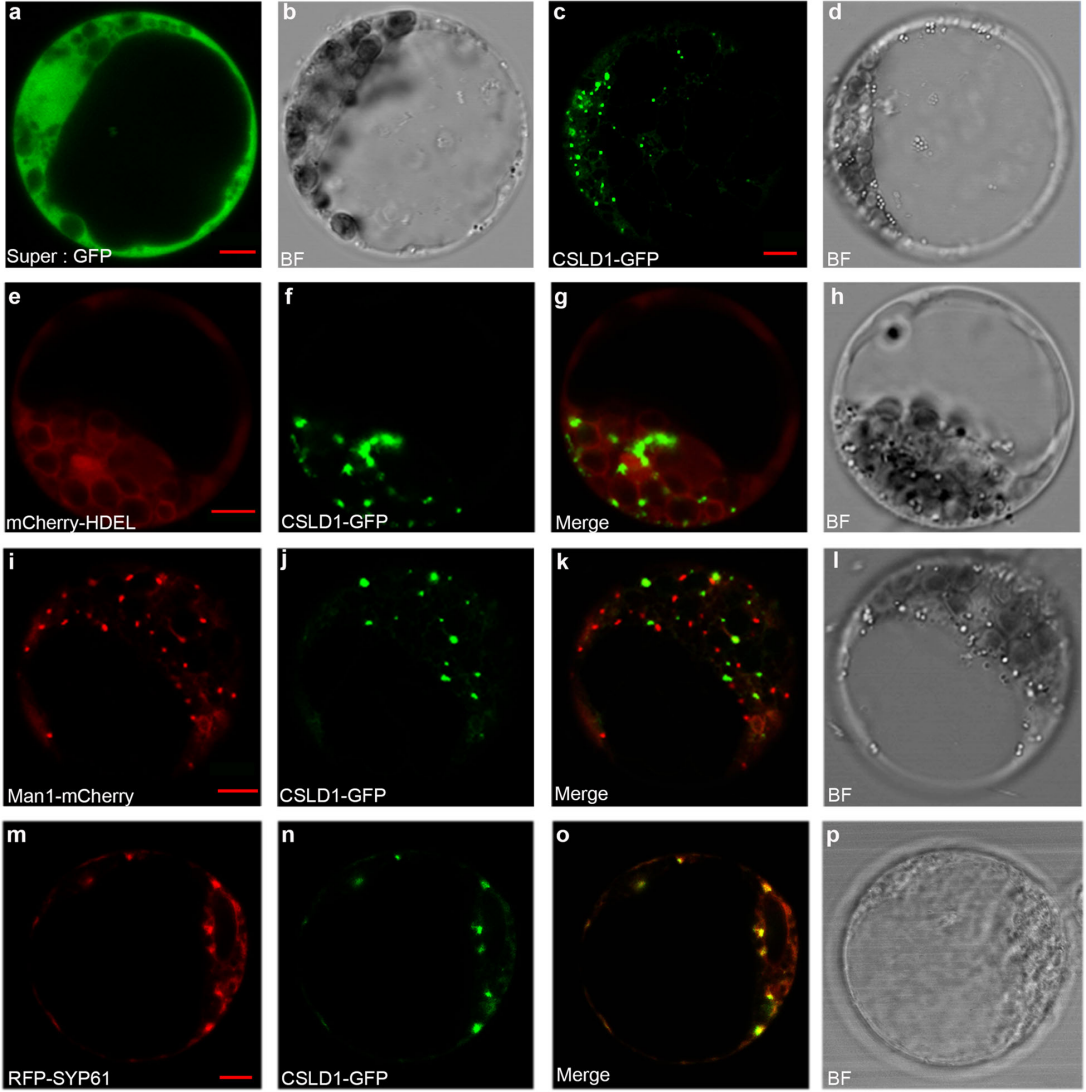
Subcellular localization of ZmCSLD1. **a**–**b** Empty *Super:GFP* vector expression observed by fluorescence (**a**) and bright-field (**b**; BF) microscopy. **c**–**d** CSLD1-GFP fusion protein expression. **e**–**h** The co-expression of the ER marker *mCherry-HDEL* and the CSLD1-GFP fusion protein (**f**), Merge (**g**) and BF (**h**) are shown. **i**–**l** The co-expression of the *cis*-Golgi marker *Man1-mCherry* (**i**) and the CSLD1-GFP fusion protein (**j**), Merge (**k**) and BF (**l**) are shown. **m**–**p** The co-expression of the *trans*-Golgi marker *RFP-SYP61* (**m**) and the CSLD1-GFP fusion protein (**n**), Merge (**q**) and BF (**p**) are shown. Scale bar = 5 μm

To examine the subcellular localization of ZmCSLD1, we transiently expressed *pSuper:ZmCSLD1-GFP* in maize protoplast cells under the blank control of *pSuper:GFP* and co-transformed it with *mCherry-HDEL-RFP* (endoplasmic reticulum (ER) marker), *Man1-mCherry-RFP* (*cis*-Golgi marker) and *RFP-SYP61* (*trans*-Golgi marker) as the controls for subcellular localization (Fig. 3a-p). A punctate pattern of GFP signals was observed in the protoplasts when *ZmCSLD1-GFP* was expressed (Fig. 3c, f and n), and this pattern was different from the ER marker location pattern (Fig. 3e). The partial overlap of ZmCSLD1-GFP with the *cis*-Golgi marker and complete merging with the *trans*-Golgi marker indicate that ZmCSLD1 localizes to the *trans*-Golgi (Fig. 3k and e); this finding is consistent with the well-known subcellular localization of its orthologues in rice (OsCSLD4) and *Arabidopsis* (AtCSLD5) [21, 37].

In previous studies, xylan and homogalacturonan synthase activity were reduced in *Atcsld5* [21], and the content of xylan, cellulose and homogalacturonan were reduced in *Oscsld4* [37]. These results suggest that *Zmcsld1* might participate in the cell wall polysaccharides biosynthesis due to their protein identity. To test the association between *ZmCSLD1* and cell wall compositions, the cell wall monosaccharide composition and cellulose content of the ear leaves in *ZmCSLD1*^*W22*^ and *Zmcsld1*^*W22*^ were measured at 7 days after flowering. Compared with *ZmCSLD1*^*W22*^, *Zmcsld1*^*W22*^ showed a significant increase of mannose and glucose content, but a decrease tendency in xylose, galactose and cellulose content (Additional file 11: Table S5). These results confirmed that *ZmCSLD1* played an important role in the cell wall polysaccharide biosynthesis.

### *ZmCSLD1* has pleiotropic and heterogeneous effects

**Fig. 4:**
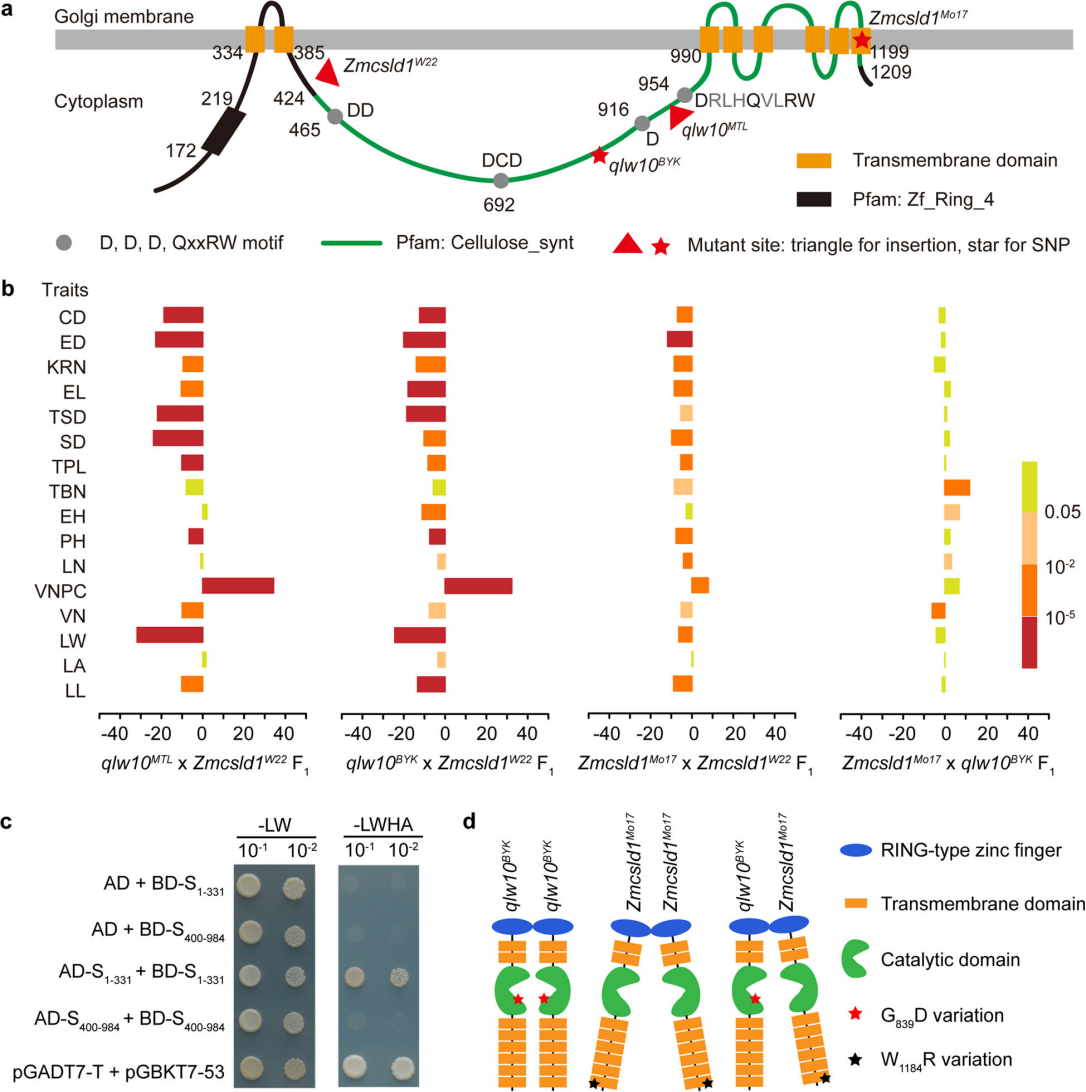
*ZmCSLD1* regulates plant architecture, and the effects of *ZmCSLD1* vary among different alleles. **a** Schematic diagram of the domains and the location of causative alleles in *Zmcsld1*. The protein domains were predicted according to the descriptions of Zeng and Keegstra [92] and Hunter et al. [36]. **b** The pleiotropic effects of *Zmcsld1* by comparing four *Zmcsld1*-combined F_1_ hybrids with their *ZmCSLD1*-combined F_1_ hybrids, respectively. The significance levels of the differences analysed by Student’s t-test (n = 30) are indicated in different colours. Traits abbreviations are listed in Additional file 2: Table S2. **c** Yeast two-hybrid system showing the interactions between AD-S_1-331_ and BD-S_1-331_. pGADT7-T/pGBKT7-53 was used as a positive control. **d** Schematic diagram of the intragenic complementation between *qlw10*^*BYK*^ and *Zmcsld1*^*Mo17*^

To validate that *ZmCSLD1* is also the causal gene that affects the other eight traits, 16 traits were examined in *qLW10*^*MTL*^ and *qlw10*^*MTL*^, *qLW10*^*BYK*^ and *qlw10*^*BYK*^, *ZmCSLD1*^*W22*^ and *Zmcsld1*^*W22*^ and *ZmCSLD1*^*Mo17*^ and *Zmcsld1*^*Mo17*^ (Additional file 12: Figure S7). Twelve traits showed large differences between *qLW10*^*MTL*^ and *qlw10*^*MTL*^, and the *qlw10*^*MTL*^ allele conferred overall decreases in these traits of 8.99% (veinlet number, VN) to 33.47% (LW). The same QTL at the *ZmCSLD1* locus was identified for each of the nine common traits in the LEE-12 × MT03-1 F_2_ population, which further indicates the pleiotropic effects of *ZmCSLD1*. Furthermore, 10 of the traits, including narrow leaves, dwarf plants, and thin stems, showed consistent patterns in all the lines with the loss-of-function alleles, whereas some traits showed significant differences between *qLW10*^*BYK*^ and *qlw10*^*BYK*^ and between *ZmCSLD1*^*W22*^ and *Zmcsld1*^*W22*^ but not between *qLW10*^*MTL*^ and *qlw10*^*MTL*^ and *ZmCSLD1*^*Mo17*^ and *Zmcsld1*^*Mo17*^. The tassel branch number (TBN), for example, which is determined by the branching ability of the shoot apical meristem, varied in the different genomic backgrounds. Even for the traits with striking effects of the loss-of-function alleles, the effects varied among the different alleles, e.g., the effects of LW ranging from 17 to 42% (Additional file 12: Figure S7). This phenomenon may be due to the heterogeneous effects of different alleles because the causal sites occurred in different protein domains (Fig. 4a). Both *qlw10*^*MTL*^ and *Zmcsld1*^*W22*^ contained large insertions before the “D,D,D,QXXRW” motif and transmembrane domains that were predicted to lead to a complete loss of the ZmCSLD1 function, whereas the missense mutations in the “D,D,D,QXXRW” motif of *qlw10*^*BYK*^ (G839D) and in the last transmembrane domain of *Zmcsld1*^*Mo17*^ (W1184R) had weak effects on the traits.

To further confirm the pleiotropic and heterogeneous effects of *ZmCSLD1*, the 16 traits were also investigated in four F_1_ hybrids, including the three pairs described above that were crossed with Zmcsld1^W22^ and two other combinations, namely, *Zmcsld1*^*Mo17*^ × *qlw10*^*BYK*^ and *ZmCSLD1*^*Mo17*^ × *qLW10*^*BYK*^ (Additional file 8: Table S4). As expected, the performance of all the measured traits in all three F_1_ hybrid crosses between the strongest allele and three other alleles supported the pleiotropic and heterogeneous effects of *ZmCSLD1*. Unexpectedly, the defective phenotype for most traits in the *Zmcsld1*^*Mo17*^ × *qlw10*^*BYK*^ F_1_ hybrid was completely complemented (Fig. 4b), similar to the phenomenon of intragenic complementation in the cellulose synthase gene in *Arabidopsis AtCESA3* [38]. A further comparison of the LW among genotypes in six F_2_ populations indicated that the effect of the *qlw10*^*MTL*^ allele was comparable to the effect of the *Zmcsld1*^*W22*^ allele because both alleles produced an abnormal ZmCSLD1 protein, whereas the *qlw10*^*BYK*^ and *Zmcsld1*^*Mo17*^ alleles had weaker effects (Additional file 9: Figure S5). In addition, the leaves of the individuals with heterozygous (C/A) genotypes were significantly wider than the leaves of the individuals with both homozygous genotypes (C/C and A/A) in the *Zmcsld1*^*Mo17*^ × *qlw10*^*BYK*^ F_2_ population (Additional file 9: Figure S5A); this finding strongly supports the *Zmcsld1*^*Mo17*^ × *qlw10*^*BYK*^ F_1_ intragenic complementation.

To test whether ZmCSLD1 could form a homodimer at molecular level, we cloned two fragments, S_1-331_ from 1 to 331 amino acids and S_400-984_ from 400 to 984 amino acids of ZmCSLD1, into both pGBKT7-BD and pGADT7-AD vectors. The yeast two hybrid analysis indicated that segment S_1-331_ could interact with itself, but segment S_400-984_ couldn’t (Fig. 4c). In addition, the mutation of *qlw10*^*BYK*^ occurs in the cytoplasmic domain of ZmCSLD1, which possesses glycosyltransferase activity, and the *Zmcsld1*^*Mo17*^ mutation is located in the last transmembrane domain of ZmCSLD1. Taken together, we raised a model to explain the intra-genic complementation between *qlw10*^*BYK*^ and *Zmcsld1*^*Mo17*^ (Fig. 4d). Both catalytic and transmembrane domains are necessary to the function of ZmCSLD1. Either mutation in these two domains in homologous lines will weaken the function of *ZmCSLD1*, while the function of *ZmCSLD1* will be complemented in F_1_ hybrids of two mutation alleles in different domains.

### Decreased cell division rate contributes to a reduced organ size

**Fig. 5:**
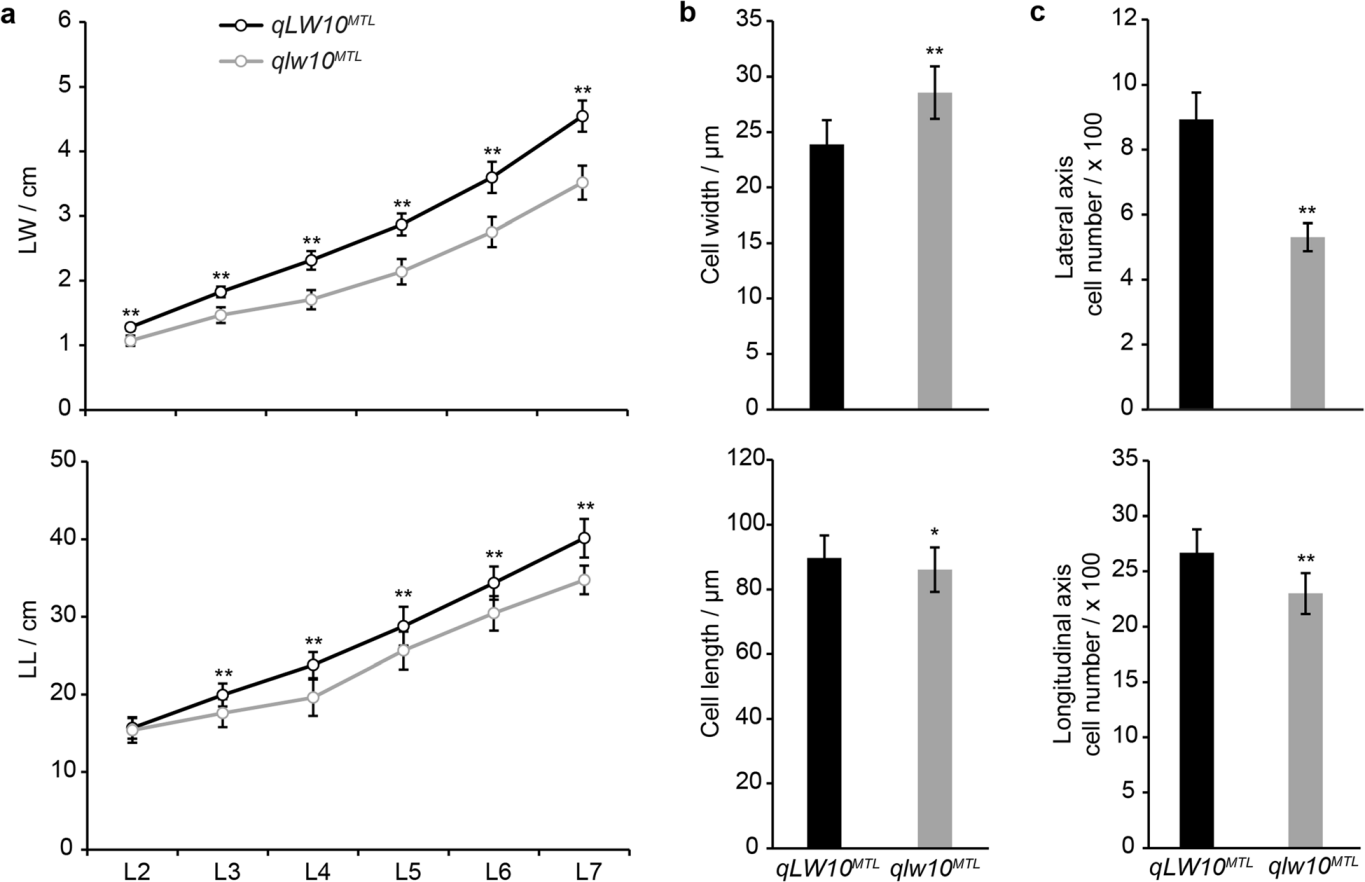
ZmCSLD1 regulates LW by cell division. **a** Comparison of LW and length between *qLW10*^*MTL*^ and *qlw10*^*MTL*^ of the six unfolded leaves (L2–7) from 40-day-old seedlings. **b**, **c** Comparison of the abaxial epidermal surface cells of the fourth middle leaf blade from 40-day-old seedlings. Fifty normal cells per plant were measured by ImageJ for the average cell width and length (**b**), and the number of cells in the lateral and longitudinal axis (**c**) of the leaf blades was estimated by dividing the average LW by the cell width and the average LL by the cell length, respectively. The data are shown as the mean ± SD (n = 30). **P < 0.01 (Student’s t-test)

Cell division and expansion are the primary ways of plant growth. The relationship between *ZmCSLD1* and cell division or expansion raises the question of whether *ZmCSLD1* has pleiotropic effects on the size of multiple organs in maize. To investigate the relationship between organ size and *ZmCSLD1*, the LL and LW of all unfold leaf blades were measured to assess the leaf morphology of 40-day-old seedlings (Fig. 5a). An increasing difference between *qLW10*^*MTL*^ and *qlw10*^*MTL*^ was observed for LW after the second leaf stage and for LL after the third leaf stage, and the fourth LW and length of *qLW10*^*MTL*^ reached 1.36 and 1.21 times the LW and LL of *qlw10*^*MTL*^, respectively. This result was highly consistent with the morphology of mature leaves (Fig. 2a and b). To address the relationship between LW and cell division or cell expansion, the cell size was quantified and the cell number was calculated for the fourth leaf, which had the greatest variation in the leaf stages (Additional file 13: Figure S8). Compared with *qLW10*^*MTL*^, cell width increased by 19.52% in *qlw10*^*MTL*^, whereas cell length decreased by 4.44% (Fig. 5b); the cell number in both the lateral and longitudinal axes was notably reduced by 40.55% and 13.79%, respectively (Fig. 5c). Similarly, all of three other alleles, *qlw10*^*BYK*^, *Zmcsld1*^*W22*^, and *Zmcsld1*^*Mo17*^*,* showed significant decrease in LW and cell number in the lateral axes of the third leaf from 20-day-old seedlings (Additional file 14: Figure S9). These results imply that the narrowing of the *qlw10*^*MTL*^ leaf was mainly caused by a decrease in cell number due to a reduction in cell division.

**Fig. 6:**
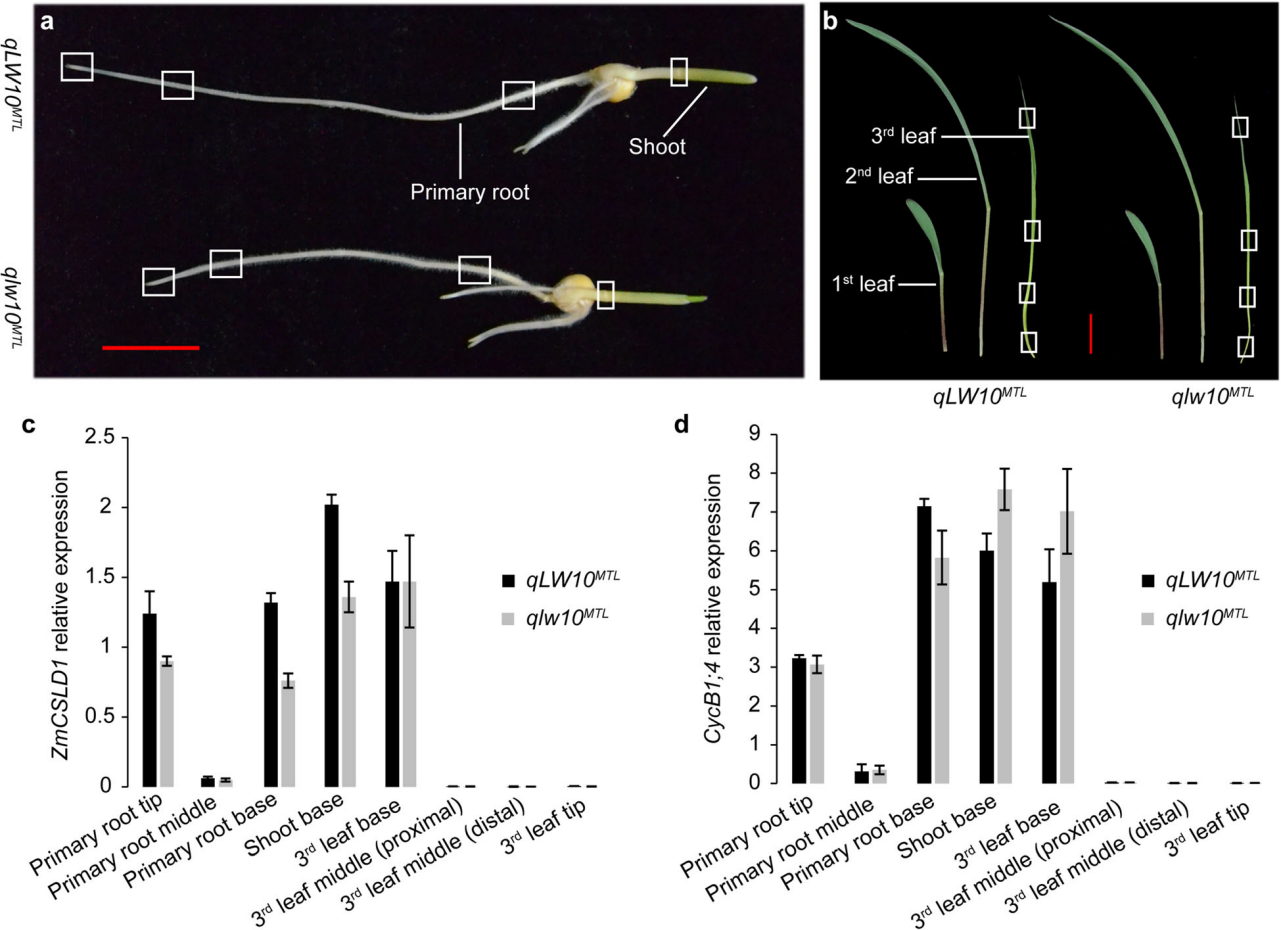
Expression patterns of *ZmCSLD1* and *CycB1;4* in root, shoot and leaf tissues in *qLW10*^*MTL*^ and *qlw10*^*MTL*^. **a**–**b** Positions of primary roots and shoot from 5-day-old seedlings (**a**) and the third leaf tissue from 10-day-old seedlings (**b**), which are indicated by white frame, were sampled for expression analysis. Scale bar = 2 cm. **c**–**d** Relative expression of *ZmCSLD1* (**c**) and *CycB1;4* (**d**) in eight tissues corresponding to (**a**) and (**b**) in *qLW10*^*MTL*^ and *qlw10*^*MTL*^ were detected by qRT-PCR, data are shown as the mean ± SE (n = 3)

In addition, based on the expression levels of *ZmCSLD1* in 13 tissues at various developmental stages [39–43], *ZmCSLD1* has been determined to be expressed in most maize tissues (Additional file 15: Figure S10). *ZmCSLD1* expression was high in the ear primordia, tassel primordia, shoot apical meristem, third leaf base and ovule but was low in the root and embryo at 10 days after pollination (DAP), in the endosperm at 6 DAP, and in the seeds at 3 DAP, and no expression was detected in the third leaf tip, pre-emergence tassel, silk or anthers (Additional file 15: Figure S10A). In addition, *ZmCSLD1* was highly expressed in the early stage of seed development, and dropped successively during the seed maturation process (Additional file 15: Figure S10B), which corresponds to a phase of mitotic cell proliferation during maize embryogenesis and endosperm development [40, 44, 45]. To further validate the association of *ZmCSLD1* with cell proliferation, we sampled three root tissues and shoot tissue from 5-day-old seedlings, and four leaf tissues of the third leaf tissues from 10-day-old seedling in *qLW10*^*MTL*^ and *qlw10*^*MTL*^ (Fig. 6a and b). As expected, *ZmCSLD1* were highly expressed in primary root tip, shoot base and the third leaf base tissues, and expressed at low levels in primary root middle, third leaf middle (proximal and distal) and tip (Fig. 6c). The expression pattern of *ZmCSLD1* was highly consistent with that of three cell circle related genes *CycB1;4*, *CycD3;1b* and histone *H2B* which expressed during G2/M transition, G1/S transition and S phase in cell cycle, respectively [46, 47] (Fig. 6d; Additional file 16: Figure S11A and B). Specially, the expression level and pattern of *ZmCSLD1* was most similar to *CycB1;4* (*r* = 0.91), suggesting that *ZmCSLD1* may also be expressed in the G2/M transition-phase during cell division. Additionally, *ZmCSLD1* was also highly expressed in primary root base tissues which might be caused by the development of lateral roots. Collectively, *ZmCSLD1* was highly expressed in the early stages during rapid growth in all tissues, whereas this expression was low in the mature stages (Fig. 6c; Additional file 15: Figure S10B), which is consistent with the function of *ZmCSLD1* that relates to cell division and development.

## Discussion

### *ZmCSLD1* participates in the cell wall formation during cell division

Organ size growth is a complex process that consists of cell division and expansion. The cell wall is an integral structure in plant cells and mainly comprises cellulose, hemicellulose and pectin. These components play essential roles in plant cell division and expansion, plant growth and, consequently, morphogenesis [48]. There are ten subfamilies in the cellulose synthase (CESA) superfamily according to protein sequence similarity, including nine cellulose synthase-like (CSL) families and one CESA family. These subfamilies contain a “D,D,D,QXXRW” motif thought to catalyse the synthesis of the β-glycosyl unit structure of cellulose and hemicellulose [49–51]. All CESA proteins localize to the plasma membrane to synthesize cellulose on the cell surface [52, 53]. Most CSL proteins, such as AtCSLA9, AtCSLC4, BdCSLF6, HvCSLH1 and AtCSLD5, accumulate in the Golgi, where they are thought to produce hemicellulose and pectin polysaccharides for subsequent vesicular secretion to the extracellular space [21, 54–56]. Heterologous expression has demonstrated that the CSLA family is related to β-mannan or glucomannan polysaccharide synthesis. CSLC proteins function in assembling the β-1,4 glucan backbone of xyloglucan, and the grass-specific families of CSLF and CSLH encode the mixed linkage glucan synthases [55, 57–59]. The other CSL families, namely, CSLB, CSLD, CSLE, CSLG and CSLJ, also likely participate in synthesizing hemicellulose polysaccharides, although it is still unclear which products they synthesize.

Among the CSL families, the CSLD proteins display the greatest amino acid sequence similarity to the CESA family [50]; therefore, the CSLD proteins were predicted to have similar functions in synthesizing cell wall components. This prediction was confirmed by the finding that the CSLD proteins participate in the synthesis of cell wall polysaccharides, such as non-crystalline cellulose and xyloglucan [21, 37, 60, 61]. Our finding that ZmCSLD1 is a *trans*-Golgi protein is consistent with the subcellular localization of its orthologues AtCSLD5 and OsCSLD4; this finding suggests that ZmCSLD1 is likely to participate in the synthesis of hemicelluloses and non-crystalline cellulose in the Golgi apparatus, or cellulose with the assembly of CSLD proteins in the Golgi followed by transport to the plasma membrane [21, 37, 60, 62–64]. In addition, the cell wall composition changed in *Zmcsld1*, especially a decrease tendency in the main components of cell wall backbone, xylose and cellulose content (Additional file 15: Table S5). These results, together with a previous study by Hunter et al. [36], suggest that *ZmCSLD1* participates in the cell wall polysaccharide biosynthesis of cell plate during cell division. Whereas, the nature of the function of ZmCSLD1 as an enzyme, including its substrates and products, remains to be identified.

### *ZmCLSD1* affects cell division and organ size and has pleiotropic effects

Pleiotropic effects, which refer to one gene that can affect many traits, have been identified in many species [65]. These effects play an important role in development and evolution because of the complexity of the biochemical and developmental networks [66]. Pleiotropic effects often cause genetic correlations in bi- or multi-parent-derived populations [6, 8]. In our study, nine traits with QTL identified at the *qLW10* locus had a moderate to high correlation with one another (*r* = 0.44–0.94, F_2:3_ population) (Fig. 1b), which differs from other studies [6–11]. For example, the correlation between LW and LL was 0.62 in our F_2:3_ population but only 0.08 in an NAM population [10]. In addition, the NILs and mutants of *ZmCSLD1* showed pleiotropic effects (Additional file 12: Figure S7), which also confirms that *ZmCSLD1* underlies the pleiotropic effects of *qLW10*.

Pleiotropic genes often participate in transcription regulation, hormone signalling, ubiquitin-mediated proteolysis and cell wall biosynthesis pathways, which control organ size by regulating cell proliferation [5, 67]. The size and growth of organs are determined by the characteristics of their cells, such as their width, length and number. Because *ZmCSLD1* encodes an enzyme in cell wall biosynthesis, it is interesting to investigate whether the pleiotropic effects of *ZmCSLD1* due to organ size variation are caused by cell division or expansion. In the current study, the reduction in cell number in both the lateral and longitudinal leaf axes was far more pronounced than the reduction in cell size (Fig. 5b and c; Additional file 14: Figure S9). Thus, the reduction in cell number appears to be the main cause of the narrow organ phenotype, which is highly consistent with the results of Hunter et al. [36]. Furthermore, *ZmCSLD1* was highly expressed in immature tissues, such as in the root tip, immature leaf base, ear primordia, shoot apex and tassel primordia and young-embryo endosperm and seed (Fig. 6c; Additional file 15: Figure S10A), but the transcript was nearly undetectable in mature tissues (Fig. 6c; Additional file 15: Figure S10B). Similar expression patterns of its orthologues in *Arabidopsis* and rice were validated by a β-glucuronidase (GUS) expression from the *AtCSLD5* and *OsCSLD4* promoters, respectively [21, 37]. The common characteristics of these tissues in which *ZmCSLD1* is highly expressed is that these tissues are regions of rapidly dividing cells. In addition, *OsCSLD4* is specifically expressed during the M phase of the cell cycle and the *AtCSLD5* functions in the cell plate formation period, both of which are critical phases in mitosis [68, 69]. The sequence conservation of *ZmCSLD1* with *OsCSLD4* and *AtCSLD5* strongly supports the role of *ZmCSLD1* in cell division.

### Mechanism of intragenic complementation between different *ZmCSLD1* alleles

The recovery of the narrow-leaf phenotype in the heterozygotes (Fig. 4b) indicates that the *Zmcsld1*^*Mo17*^ and *qlw10*^*BYK*^ alleles of the *ZmCLSD1* gene exhibit intragenic complementation. Intragenic complementation has been reported in plants, e.g., among the pattern formation gene *GNOM* [70], the abscisic acid biosynthesis gene *ABA2* [71], the cytokinin receptor gene *WOL/CRE1* [72], the brassinosteroid receptor gene *BRI1* [73], and the cellulose biosynthesis genes *AtCESA1* [74] and *AtCESA3* [38]. The common characteristics of these genes is that they encode proteins with multiple functional domains or proteins that form homomeric or higher order homomultimeric complexes. For example, AtCESA1 and AtCESA3 are a part of the large cellulose synthase complex that contains approximately 36 CESA polypeptides in *Arabidopsis* and that is divided into six rosette subunits, each of which consists of three CESA isoforms [53, 54]. The three CESA isoforms form cellulose synthase complexes through various heterodimeric and/or homodimeric combinations to synthesize the cellulose microfibrils of the cell wall [75–77]. Therefore, these CESA genes can complement one another when the product of one allele functionally compensates for the product of a second allele in the same complex.

Similarly, the proteins of the CLSD family also likely form a heterodimeric or homodimeric enzyme complex that functions in generating non-crystalline cellulose or the β-1,4 glucan backbone of hemicellulose [78]. Notably, the mutations in *qlw10*^*BYK*^ and *Zmcsld1*^*Mo17*^ result in different amino acid residues in the “D,D,D,QXXRW” motif (G839D) and the last transmembrane domain (W1184R), respectively (Fig. 4a). Thus, the mechanism of intragenic complementation in the F_1_ hybrids between *qlw10*^*BYK*^ and *Zmcsld1*^*Mo17*^ by the formation of the homodimers of the two mutant alleles that compensate for the defective cytosolic catalytic domain and the disabled transmembrane domain in the same complex, which consequently reduces or recovers the narrow-leaf phenotype in the heterozygotes (Fig. 4b–d). However, further studies are necessary to confirm how many ZmCLSD1 subunits are present in the complex and to determine whether other protein subunits are involved.

## Conclusion

Our results show that *qLW10* was resolved to be *ZmCSLD1* through map-based cloning. *ZmCSLD1* encodes an enzyme in cell wall biosynthesis and controls organ size by altering cell division, which results in pleotropic effects on multiple traits in maize. The subcellular localization of ZmCSLD1 in the *trans*-Golgi shows that ZmCSLD1 participates in cell wall polysaccharides formation during cell division. In addition, ZmCSLD1 may form a homomeric or higher order homomultimeric complex to catalyse polysaccharide biosynthesis because of intragenic complementation.

## Methods

### Plant materials and growth conditions

Two maize double haploid lines, MT03-1 with a small plant architecture and LEE-12 with a large plant architecture, were used to develop an F_2_ population including 197 individuals. A heterozygous inbred family, BYK-HIF, was also used, which was derived from a By815 × K22 recombinant inbred line population. A pair of NILs, *ZmCSLD1*^*MO17*^ and *Zmcsld1*^*MO17*^, were developed from a line in a Mo17 × X26–4 (*Zea mays ssp. mexicana*) BC_2_F_5_ population by backcrossing once with Mo17 and selfing four times. A UniformMu line (UFMu-04904) was ordered from the Maize Genetics Cooperation Stock Center and backcrossed twice with W22 to reduce the background, which generated *ZmCSLD1*^*W22*^ and *Zmcsld1*^*W22*^. The plants were grown in the fields for QTL mapping, fine mapping and allelism tests.

### Construction of a genetic linkage map and QTL mapping

All 197 F_2_ individuals and F_2:3_ families in the LEE-12 × MT03-1 F_2_ population, along with both parents, were grown in Beijing (40°N, 116°E), China, in 2012 and Shenyang (42°N, 123°E), China, in 2013, respectively. All F_2_ individuals and their parents were genotyped by using the MaizeSNP3K subset (3072 SNPs) of the Illumina MaizeSNP50 BeadChip [79]. SNP genotyping was performed on the Illumina GoldenGate SNP genotyping platform [80] at the National Maize Improvement Center of China, China Agricultural University. The quality of each SNP was checked manually, and poor-quality SNPs were excluded. Using 767 high-quality polymorphic SNPs, a genetic map of 1833.66 cM with an average interval of 2.42 cM between adjacent markers was constructed by R/qtl. QTL mapping for 11 measured traits (Additional file 1: Table S1; Additional file 2: Table S2) in both the F_2_ and F_2:3_ populations was performed by using composite interval mapping [81] that was implemented in Windows QTL Cartographer 2.5 [82]. The threshold logarithm of odds (LOD) value to declare the putative QTL was estimated by permutation tests with a minimum of 1,000 replicates at a significance level of *p* < 0.05 [83]. The confidence interval of the QTL position was determined by using the 1.5-LOD support interval method [84].

### Positional cloning of *qLW10*

Fine mapping of *qLW10* was based on 3,292 BC_2_F_1_ and 669 BC_3_F_1_ plants bred from the backcross between MT03-1 and LEE-12 with MT03-1 as the recurrent parent. Additionally, an F_6_ family, BYK-HIF, in the By815 × K22 RIL population that had heterozygous alleles at the *qLW10* locus and that varied in LW was also used to fine map *qLW10* based on 720 F_2_ plants descended from BYK-HIF. The markers used for positional cloning by using the two populations are listed in Additional file 17: Table S6.

### Genotyping of various *Zmcsld1* alleles

To determine the causal variants of the four *Zmcsld1* alleles, full-length *ZmCSLD1* was sequenced from two pairs of NILs, *qLW10*^*MTL*^/*qlw10*^*MTL*^ and *qlw10*^*BYK*^/*qLW10*^*BYK*^, and two pairs of mutants and their wild type, *Zmcsld1*^*MO17*^/*ZmCSLD1*^*MO17*^ and *ZmCSLD1*^*W22*^/*Zmcsld1*^*W22*^. The genotype of *Zmcsld1*^*W22*^ was confirmed with the Mu-transposon primer TIR6 and gene-specific primer CSLD1-P3. A list of primers for this genotyping is given in Additional file 17: Table S6. Furthermore, four *Zmcsld1* alleles and their wild-type counterparts were genotyped by using the MaizeSNP6K subset (5,259 SNPs) of the Illumina MaizeSNP50 BeadChip to check their genetic backgrounds.

### Allelism tests

Ten F_1_ hybrids were generated from eight lines with different *ZmCSLD1* alleles to evaluate the allelic effects as described in Additional file 8: Table S4. All the hybrids were planted in a randomized complete block design with two replications in Sanya (18°N, 110°E), China, in 2015. Each hybrid was grown in a two-row plot with 5-m rows and 0.67 m between rows. The 16 traits were measured in at least 30 plants per hybrid as described in Additional file 2: Table S2. In addition, ~ 120 individuals of six F_2_ populations, including *qlw10*^*MTL*^ × *qlw10*^*BYK*^, *qlw10*^*MTL*^ × *Zmcsld1*^*W22*^, *qlw10*^*BYK*^ × *Zmcsld1*^*W22*^, *Zmcsld1*^*Mo17*^ × *qlw10*^*MTL*^, *Zmcsld1*^*Mo17*^ × *qlw10*^*BYK*^ and *Zmcsld1*^*Mo17*^ × *Zmcsld1*^*W22*^, were planted in Sanya (18°N, 110°E) in 2015. These individuals were genotyped by SNP and Mu-transposon insertion markers (Additional file 17: Table S6) and were phenotyped for LW, LL, PH, EH (Additional file 2: Table S2).

### Construction of the phylogenetic tree

The predicted protein sequences of ZmCSLD1 orthologues were searched by using BLAST against the UniProt database (http://www.uniprot.org/blast/) for sequences with alignment scores > 3,870, and one sequence in each species was kept for analysis. These protein sequences were aligned by using MUSCLE [85], and the aligned sequences were used to construct the phylogenetic tree by using the maximum likelihood method with 1,000 bootstrap replications in MEGA version 6.0 [86].

### Yeast two-hybrid assays

Two seqments of ZmCLSD1 (S_1–331_: from 1 to 331 amino acids, and S_400–984_: from 400 to 984 amino acids of ZmCSLD1 protein sequence) were amplified by using primers AD-GS1, BD-GS1 and AD-GS2, BD-GS2 (Additional file 17: Table S6), respectively. And the PCR products were cloned into the pGBKT7-BD and the pGADT7-AD vector via EcoRI and BamHI restriction sites using a Hieff Clone one-step PCR cloning kit (Yisheng, China), respectively. Four different combinations were cotransformed into the yeast strain AH109. Yeast cells harboring both pGADT7-AD and pGBKT7-BD vectors were selected on SD/−Leu/−Trp (−LW) medium. Interactions of all different combinations were tested on SD/−Leu/−Trp/−His/−Ade (-LWHA) medium (Clontech). pGADT7-T/pGBKT7–53 was used as a positive control. Plates were incubated at 30 °C for 5 days.

### Subcellular localization

The coding sequence of *ZmCSLD1* was amplified by using a cDNA template from 5-day-old B73 seedlings. The PCR product was amplified by using primer rCSLD1–03 (Additional file 17: Table S6) and cloned into the SUP1300 vector at the XbaI and SpeI sites to encode a ZmCSLD1-GFP fusion protein with the Super promoter. Maize protoplasts were obtained as described by Burdo et al. [87], except that B73 was used instead of B73 × Mo17 F_1_. Plasmids (15 μg) were transformed into protoplasts and incubated for 16 to 20 h in the dark at 25 °C before monitoring their GFP expression by using confocal microscopy (Zeiss 710, Germany).

### Measurement of leaf and cell size

To measure leaf size and cell number, the NILs, *qLW10*^*MTL*^ and *qlw10*^*MTL*^, were grown in the field, and the completely extended fourth leaf from 40-day-old seedlings was sampled. Simultaneously, the LL and LW of the six unfolded leaves (L2–7) were measured. For the remaining *qlw10*^*BYK*^/*qLW10*^*BYK*^, and two additional mutants and their wide type, *Zmcsld1*^*MO17*^/*ZmCSLD1*^*MO17*^, and *ZmCSLD1*^*W22*^/*Zmcsld1*^*W22*^, were planted in greenhouse, and the leaf and cell size of the third leaf from 20-day-old seedlings was measured.The replication of the abaxial epidermal surface in the middle leaf was carried out as described by Moon et al. [88]. Briefly, colorless nail polish was applied to the leaf abaxial surface and allowed to dry completely, and then the dried nail polish replicas were peeled off by transparent adhesive tape and pasted to glass slides. Leaf epidermal cells were observed and photographed by using light microscopy (Leica DM2000 LED, Germany) with a Leica DFC450 camera. For each sample, cell sizes were measured using ImageJ 1.45 s (ImageJ, National Institutes of Health, USA) in a 0.585 mm by 0.785 mm field, and at least 50 normal cells except stomata guard cell, smaller and irregular cells were measured per sample.

### RNA isolation and qRT-PCR

*qLW10*^*MTL*^ and *qlw10*^*MTL*^ were planted in greenhouse with 16-h-light/8-h-dark photoperiod for expression analysis. The primary root tip (5 mm, cell division region), middle (5 mm, cell elongation region), base (5 mm, root hair region), and shoot base (2 mm, including shoot apical meristem) from 5-day-old seedlings (Fig. 6a), and the third leaf base (1 cm), proximal middle (1 cm), distal middle (1 cm), and tip (1 cm) from 10-day-old seedlings (Fig. 6b) were sampled for analyzing *ZmCSLD1*, *CycB1;4*, *CycD3;1b* and histone *H2B* expression. Each tissue had three independent biological replicates with ten plants per biological replicate. Total RNA was extracted using a TianGen plant RNA extraction kit (China). First-strand cDNA was synthesized using the PrimeScript 1st Strand cDNA Synthesis kit (TaKaRa, Japan). qRT-PCR was carried out in triplicate for each sample using the SYBRGreen I kit (TaKaRa) on a 7500 Real-Time PCR System (Applied Biosystems, USA). Maize *TUBG* was used for normalization between samples [89]. Quantification of relative expression was based on the comparative threshold cycle method [90]. The primers used for qRT-PCR were listed in Additional file 17: Table S6.

### Cell wall components analysis

The ear leaves of *ZmCSLD1*^*W22*^ and *Zmcsld1*^*W22*^ were sampled at 7 days after flowering. Three independent biological replicates with five plants per biological replicate and five technical replicates for each sample were analyzed. Leaf veins were excluded before grinding to powder. Analysis of cell wall monosaccharide composition and cellulose content was carried out at the Institute of Genetics and Developmental Biology at Chinese Academy of Sciences (Beijing, China). Alcohol-insoluble residues (AIR) were prepared as previously described [37]. The Cell wall monosaccharide composition was determined with a gas chromatograph-coupled mass spectrometer (7890A-5975C, Agilent), and the crystalline cellulose content was quantified by an anthrone method as described previously [91].

## Additional files

Additional file 1: **Table S1.** Summary statistics of 11 agronomic traits in parental, F_2_ and F_2:3_ populations. (DOCX 15 kb)

Additional file 2: **Table S2.** Traits analyzed in this study. (DOCX 14 kb)

Additional file 3: **Table S3.** Summary of QTLs for 11 agronomic traits in the LEE-12 x MT03-1 F_2_ and F_2:3_ populations. (DOCX 27 kb)

Additional file 4: **Figure S1.** Positional cloning of *qLW10* using the HIF family BYK-HIF in the By815 × K22 recombinant inbred line population. (DOCX 323 kb)

Additional file 5: **Figure S2.** Characteristics of plant architecture in *qLW10*^*MTL*^ and *qlw10*^*MTL*^. (DOCX 683 kb)

Additional file 6: **Figure S3.** Characteristics of plant architecture in *qLW10*^*BYK*^ and *qlw10*^*BYK*^. (DOCX 783 kb)

Additional file 7: **Figure S4.** Schematic representation of the ZmCSLD1 protein. (DOCX 498 kb)

Additional file 8: **Table S4.** Genetic design of the allelism tests. (DOCX 14 kb)

Additional file 9: **Figure S5.** Box plots showing the four *Zmcsld1* allelic effects in six F_2_ populations. (DOCX 358 kb)

Additional file 10: **Figure S6.** Phylogenetic tree of CSLD orthologs across 32 angiosperms. (DOCX 256 kb)

Additional file 11: **Table S5.** Comparisons of cell wall monosaccharide composition and cellulose of leaf blades without leaf veins. (DOCX 16 kb)

Additional file 12: **Figure S7.** The pleiotropic effects of *Zmcsld1* estimated by comparing *Zmcsld1* and *ZmCSLD1* homologous lines among different alleles. (DOCX 114 kb)

Additional file 13: **Figure S8.** Epidermal impressions of the fourth leaf abaxial surfaces from 40-day-old seedlings. (DOCX 1468 kb)

Additional file 14: **Figure S9.** Comparisons of leaf and cell size between *ZmCSLD1* and *Zmcsld1* in other three alleles. (DOCX 311 kb)

Additional file 15: **Figure S10.** Expression pattern of *ZmCSLD1* in various tissues at different developmental stages. (DOCX 238 kb)

Additional file 16: **Figure S11.** Relative expression of histone *H2B* and *CycD3;1b* in root, shoot and leaf tissues in *qLW10*^*MTL*^ and *qlw10*^*MTL*^. (DOCX 166 kb)

Additional file 17: **Table S6.** List of primers used in this study. (DOCX 18 kb)

## Abbreviations

AIR: Alcohol-insoluble residues
CESA: Cellulose synthase
CSL: Cellulose synthase-like
DAP: Days after pollination
EH: Ear height
ER: Endoplasmic reticulum
HIF: Heterozygous inbred family
KW100: 100-kernel weight
LA: Leaf angle
LL: Leaf length
LN: Leaf number
LW: Leaf width
NAM: Nested association mapping
PH: Plant height
QTL: Quantitative trait locus
TBN: Tassel branch number
TL: Tassel length
VN: Veinlet number
