# Supplementary material for: Cellulose synthase-like D1 controls organ size in maize
Source: BMC Plant Biol. 2018 Oct 16;18:239. doi: 10.1186/s12870-018-1453-8 (PMC6192064; doi:10.1186/s12870-018-1453-8)
Supplement: Supplementary file 1 — Table S1. Summary statistics of 11 agronomic traits in parental, F2 and F2:3 populations. (DOCX 15 kb) [file 12870_2018_1453_MOESM1_ESM.docx]

**Additional file 1: Table S1.** Summary statistics of 11 agronomic traits in parental, F_2_ and F_2:3_ populations

| Trait^a^ | LEE-12 (♀) | MT03-1 (♂) | F_2_ | | | F_2:3_ | | | |  |
| --- | --- | --- | --- | --- | --- | --- | --- | --- | --- | --- |
|  | Mean ± SD | | Mean ± SD | Range | | Mean ± SD | | Range | |  |
| LW (cm) | 9.66 ± 0.48 | 4.49 ± 0.29 | 9.12 ± 2.1 | | 4.5–12.7 | | 9.15 ± 1.89 | | 4.7–13.3 | |
| LL (cm) | 76.01 ± 3.49 | 56.05 ± 3.56 | 74.25 ± 9.69 | | 44.6–94.5 | | 72.13 ± 8.85 | | 46–94.13 | |
| LA (°) | 36.96 ± 6.31 | 23.97 ± 4.27 | 35.03 ± 8.01 | | 15.3–58 | | 29.78 ± 4.81 | | 18.5–47.2 | |
| PH (cm) | 176.20 ± 9.15 | 150.23 ± 7.52 | 200.24 ± 28.73 | | 102–281 | | 198.91 ± 29.09 | | 93.75–256.13 | |
| EH (cm) | 63.26 ± 7.11 | 38.77 ± 7.51 | 72.19 ± 13.06 | | 36–102 | | 64.66 ± 12.34 | | 20.5–93 | |
| EL (cm) | 16.57 ± 1.04 | 11.19 ± 0.69 | 14.71 ± 3.21 | | 6.0–21.5 | | 15.89 ± 2.91 | | 7.60–22.27 | |
| ED (cm) | 32.08 ± 1.88 | 23.93 ± 1.39 | 32.75 ± 5.28 | | 19–42 | | 37.75 ± 4.11 | | 23.43–47.11 | |
| KRN (row) | 13 ± 1.04 | 10.13 ± 0.52 | 12.86 ± 2.24 | | 8.0–18.0 | | 12.58 ± 1.72 | | 8.0–18.0 | |
| CD (cm) | 21.13 ± 1.26 | 17.06 ± 1 | 21.82 ± 3.56 | | 11.21–29.44 | | 21.72 ± 2.79 | | 13.79–29 | |
| CW (cm) | 8.95 ± 1.06 | 6.23 ± 0.82 | 13.06 ± 6.04 | | 1.42–30.48 | | 14.53 ± 5.39 | | 2.82–36.43 | |
| KW100 (g) | 20.58 ± 0.72 | 23.76 ± 0.88 | 28.42 ± 6.94 | | 10.51–45.15 | | 27.67 ± 3.84 | | 16.73–37.62 | |

^a^ LW, leaf width; LL, leaf length; LA, leaf angle; PH, plant height; EH, ear height; EL, ear length; ED, ear diameter; KRN, kernel row number; CD, cob diameter; CW, cob weight; KW100, 100-kernal weight.
