# Supplementary material for: Cellulose synthase-like D1 controls organ size in maize
Source: BMC Plant Biol. 2018 Oct 16;18:239. doi: 10.1186/s12870-018-1453-8 (PMC6192064; doi:10.1186/s12870-018-1453-8)
Supplement: Supplementary file 2 — Table S2. Traits analyzed in this study. (DOCX 14 kb) [file 12870_2018_1453_MOESM2_ESM.docx]

**Additional file 2: Table S2.** Traits analyzed in this study

| Traits | Description |
| --- | --- |
| Leaf width (LW) | Leaf width of the top ear (cm) |
| Leaf length (LL) | Leaf length of the top ear (cm) |
| Leaf angle (LA) | Angle between the first leaf above the top ear and the stem (°) |
| Plant height (PH) | Height from the ground to the tip of the main tassel branch (cm) |
| Ear height (EH) | Height from the ground to the ear (cm) |
| Tassel branch number (TBN) | Number of tassel branches |
| Tassel length (TL) | Length of the main stem of the tassel (cm) |
| Stem diameter (SD) | Stem diameter of first stem below top ear (mm) |
| Tassel stem diameter (TSD) | Stem diameter of main tassel base (mm) |
| Leaf number (LN) | Number of total leaves |
| Veinlet number (VN) | Veinlet number of the top ear leaf at the widest section except for midrib |
| Veinlet number per centimeter (VNPC) | Veinlet number compared to leaf width of top ear leaf |
| Ear length (EL) | Length of the ear (cm) |
| Ear diameter (ED) | Diameter of the ear (mm) |
| Kernel row number (KRN) | Row number of kernel per circle (row) |
| Cob diameter (CD) | Diameter of the cob (mm) |
| 100-Kernel weight (KW100) | One hundred kernel weight (g) |
