# Supplementary material for: Cellulose synthase-like D1 controls organ size in maize
Source: BMC Plant Biol. 2018 Oct 16;18:239. doi: 10.1186/s12870-018-1453-8 (PMC6192064; doi:10.1186/s12870-018-1453-8)
Supplement: Supplementary file 3 — Table S3. Summary of QTLs for 11 agronomic traits in the LEE-12 x MT03-1 F2 and F2:3 populations. (DOCX 27 kb) [file 12870_2018_1453_MOESM3_ESM.docx]

**Additional file 3: Table S3.** Summary of QTLs for 11 agronomic traits in the LEE-12 x MT03-1 F_2_ and F_2:3_ populations

| Trait^a^ | Pop | Chr | QTL^b^ | Peak (cM)^c^ | Left marker | Right marker | Genetic interval (cM) | Physical interval (Mb)^d^ | LOD^e^ | A^f^ | D^g^ | R^2^ (%)^h^ |
| --- | --- | --- | --- | --- | --- | --- | --- | --- | --- | --- | --- | --- |
| LW | F_2_ | 2 | *qLW2* | 162.87 | PZE-102153048 | PZE-102175664 | 148.86–176.62 | 199.55–218.46 | 3.97 | –0.19 | 0.72 | 3.27 |
|  |  | 10 | *qLW10* | 43.78 | PZE-110010390 | PZE-110023431 | 32.95–45.33 | 8.93–34.93 | 83.86 | 2.25 | 1.97 | 75.05 |
|  | F_2:3_ | 5 | *qLW5* | 6.01 | SYN20117 | PZE-105012420 | 0–19.29 | 1.25–5.44 | 4.77 | –0.5 | –0.11 | 3.44 |
|  |  | 10 | *qlLW10* | 43.78 | PZE-110010390 | PZE-110023431 | 32.95–45.33 | 8.93–34.93 | 76.82 | 2.05 | 1.4 | 74.58 |
| LL | F_2_ | 10 | *qLL10* | 42.96 | PZE-110010390 | PZE-110023431 | 32.95–45.33 | 8.93–34.93 | 29.41 | 6.08 | 9.03 | 40.44 |
|  | F_2:3_ | 4 | *qLL4* | 38.64 | PZE-104022557 | PZE-104026267 | 29.01–61.17 | 24.98–31.31 | 5.35 | –4.09 | –0.62 | 9.68 |
|  |  | 8 | *qLL8* | 61.54 | PZE-108037749 | SYN16948 | 56.79–71.57 | 60.88–106.28 | 6.77 | 4.38 | 1.9 | 9.67 |
|  |  | 10 | *qLL10* | 43.78 | PZE-110010390 | PZE-110023431 | 32.95–45.33 | 8.93–34.93 | 24.48 | 5.8 | 5.77 | 34.46 |
| LA | F_2:3_ | 2 | *qLA2* | 33.92 | SYN6306 | SYN4735 | 4.13–25.91 | 2.79–9.44 | 4.2 | –2.12 | 0.34 | 10.84 |
|  |  | 4 | *qLA4* | 24.32 | PZE-104019988 | PZB01619.2 | 21.52–32.63 | 21.22–29.17 | 6.73 | –2.3 | 0.5 | 12.72 |
|  |  | 8 | *qLA8* | 97.49 | PZE-108075601 | PZE-108091190 | 90.53–103.94 | 131.12–148.26 | 4.28 | 0.58 | 2.31 | 7.16 |
| PH | F_2_ | 1 | *qPH1-1* | 210.04 | ZM013367-0314 | PZE-101205523 | 199.53–213.81 | 239.434–254.24 | 6.38 | 12.73 | 6.03 | 9.86 |
|  |  | 10 | *qPH10* | 41.96 | PZE-110010390 | PZE-110023431 | 32.95–45.33 | 8.93–34.93 | 23.92 | 18.34 | 22.09 | 36.15 |
|  | F_2:3_ | 1 | *qPH1-2* | 238.95 | PZE-101211251 | PZE-101222687 | 220.21–231.94 | 260.96–273.87 | 5.06 | 12.27 | 1.32 | 6.51 |
|  |  | 10 | *qPH10* | 44.52 | PZE-110010390 | PZE-110023431 | 32.95–45.33 | 8.93–34.93 | 42.28 | 26.52 | 23.5 | 57.35 |
| EH | F_2_ | 1 | *qEH1-1* | 211.04 | PZE-101203291 | PZE-101205523 | 207.03–213.81 | 250.91–254.24 | 9.69 | 7.96 | 4.36 | 18.26 |
|  |  | 10 | *qEH10* | 36.96 | SYN17100 | PZE-110018194 | 25.31–43.77 | 5.74–21.50 | 4.67 | 3.87 | 5.34 | 9.18 |
|  | F_2:3_ | 1 | *qEH1-2* | 231.25 | PZE-101211251 | PZE-101222687 | 220.21–231.94 | 260.96–273.87 | 9.85 | 6.17 | –0.97 | 13.7 |
|  |  | 3 | *qEH3* | 40.83 | SYN10329 | SYN6632 | 24.35–50.37 | 4.85–16.57 | 3.81 | –3.7 | 0.67 | 4.69 |
|  |  | 10 | *qEH10* | 44.52 | PZE-110010390 | PZE-110023431 | 32.95–45.33 | 8.93–34.93 | 28.56 | 9.01 | 7.95 | 37.84 |
| EL | F_2_ | 4 | *qEL4* | 157.83 | PZE-104140817 | PZE-104144719 | 154.55–159.71 | 229.18–233.54 | 4.29 | 1 | –1.07 | 6.39 |
|  |  | 10 | *qEL10* | 40.96 | PZE-110010390 | PZE-110018194 | 32.95–43.77 | 8.93–21.50 | 30.83 | 2.52 | 2.62 | 46.15 |
|  | F_2:3_ | 3 | *qEL3* | 154.89 | PZE-103149619 | SYN31522 | 150.31–170.78 | 203.76–217.56 | 4.99 | 0.86 | –0.87 | 6.64 |
|  |  | 10 | *qEL10* | 43.78 | PZE-110010390 | PZE-110023431 | 32.95–45.33 | 8.93–34.93 | 50.58 | 2.39 | 2.74 | 59.18 |
| ED | F_2_ | 10 | *qED10* | 44.52 | PZE-110010390 | PZE-110023431 | 32.95–45.33 | 8.93–34.93 | 24.16 | 4.1 | 4.38 | 40.7 |
|  | F_2:3_ | 7 | *qED7* | 60.56 | PZE-107067144 | SYN22401 | 52.55–66.51 | 123.85–134.10 | 4.4 | 1.35 | 1.16 | 6.79 |
|  |  | 10 | *qED10* | 44.52 | PZE-110010390 | PZE-110023431 | 32.95–45.33 | 8.93–34.93 | 48.18 | 3.54 | 3.79 | 61.39 |
| KRN | F_2_ | 2 | *qKRN2* | 12.18 | SYN4121 | SYN4735 | 0–25.91 | 1.81–9.44 | 4.52 | –0.84 | –0.28 | 7.47 |
|  |  | 5 | *qKRN5* | 39.47 | PZA02029.19 | PZE-105024349 | 27.05–39.7 | 8.00–12.04 | 3.64 | –0.68 | 0.6 | 5.55 |
|  |  | 10 | *qKRN10* | 43.78 | PZE-110010390 | PZE-110023431 | 32.95–45.33 | 8.93–34.93 | 24.94 | 1.87 | 1.65 | 41.03 |
|  | F_2:3_ | 5 | *qKRN5* | 40.71 | SYN37537 | PZE-105032165 | 29.1–52.44 | 9.38–17.38 | 3.62 | –0.5 | 0.35 | 4.84 |
|  |  | 10 | *qKRN10* | 43.78 | PZE-110010390 | PZE-110023431 | 32.95–45.33 | 8.93–34.93 | 27.1 | 1.2 | 1.1 | 37.75 |
| CD | F_2_ | 10 | *qCD10* | 43.78 | PZE-110010390 | PZE-110023431 | 32.95–45.33 | 8.93–34.93 | 31.39 | 2.82 | 3.18 | 49.02 |
|  | F_2:3_ | 10 | *qCD10* | 44.52 | PZE-110010390 | PZE-110023431 | 32.95–45.33 | 8.93–34.93 | 53.44 | 2.54 | 2.67 | 64.63 |
| CW | F_2_ | 10 | *qCW10* | 52.73 | PZE-110010390 | PZE-110041758 | 32.95–55.79 | 8.93–79.94 | 25.08 | 4.65 | 4.91 | 45 |
|  | F_2:3_ | 2 | *qCW2* | 25.92 | PZE-102017883 | SYN4735 | 20.9–25.91 | 7.95–9.44 | 3.96 | –1.5 | –0.99 | 4.88 |
|  |  | 5 | *qCW5* | 32.11 | SYN20117 | PZE-105024195 | 0–39.46 | 1.25–11.95 | 4.11 | –1.64 | –0.9 | 5.55 |
|  |  | 10 | *qCW10* | 44.52 | PZE-110010390 | PZE-110023431 | 32.95–45.33 | 8.93–34.93 | 38.28 | 4.17 | 4.55 | 47.28 |
| KW100 | F_2_ | 1 | *qKW1* | 71.82 | SYN8490 | PZE-101061168 | 59.33–81.18 | 31.87–45.10 | 4.21 | –2.65 | 1.15 | 8.1 |
|  |  | 4 | *qKW4* | 97.03 | PZE-104046399 | SYN13970 | 83.84–97.51 | 69.47–153.63 | 3.62 | 2.47 | 1.23 | 6.86 |
|  |  | 5 | *qKW5* | 63.09 | PZE-105032165 | SYN1421 | 52.44–68.69 | 17.38–58.53 | 4.17 | 2.6 | 1.5 | 7.9 |
|  |  | 10 | *qKW10* | 57.08 | PZE-110019199 | PZE-110068110 | 44.51–66.02 | 23.21–124.53 | 4.03 | 2.03 | 2.69 | 7.4 |
|  | F_2:3_ | 1 | *qKW1* | 85.28 | PZE-101061168 | PZE-101075097 | 81.18–94.47 | 45.10–58.50 | 4.24 | –1.41 | –0.85 | 7.71 |
|  |  | 2 | *qKW2* | 88.26 | PZE-102065179 | PZE-102132750 | 71.3–97.51 | 43.20–183.39 | 4.9 | –1.1 | –1.52 | 8.64 |

^a^ Trait abbreviations are listed in Additional file 2: Table S2; ^b^ QTL names defined in this study; ^c^ The genetic position of a QTL; ^d^ Physical distance of the flanking markers according to B73 RefGen_V3; ^e^ The peak LOD value of a QTL; ^f, g^ Additive effect and dominant effect of the QTL, respectively; + and – represent alleles from LEE-12 and MT03-1, respectively; ^h^ Percentage of phenotypic variation explained by the QTL.
