# Supplementary material for: Cellulose synthase-like D1 controls organ size in maize
Source: BMC Plant Biol. 2018 Oct 16;18:239. doi: 10.1186/s12870-018-1453-8 (PMC6192064; doi:10.1186/s12870-018-1453-8)
Supplement: Supplementary file 4 — Figure S1. Positional cloning of qLW10 using the HIF family BYK-HIF in the By815 × K22 recombinant inbred line population. (DOCX 323 kb) [file 12870_2018_1453_MOESM4_ESM.docx]

**
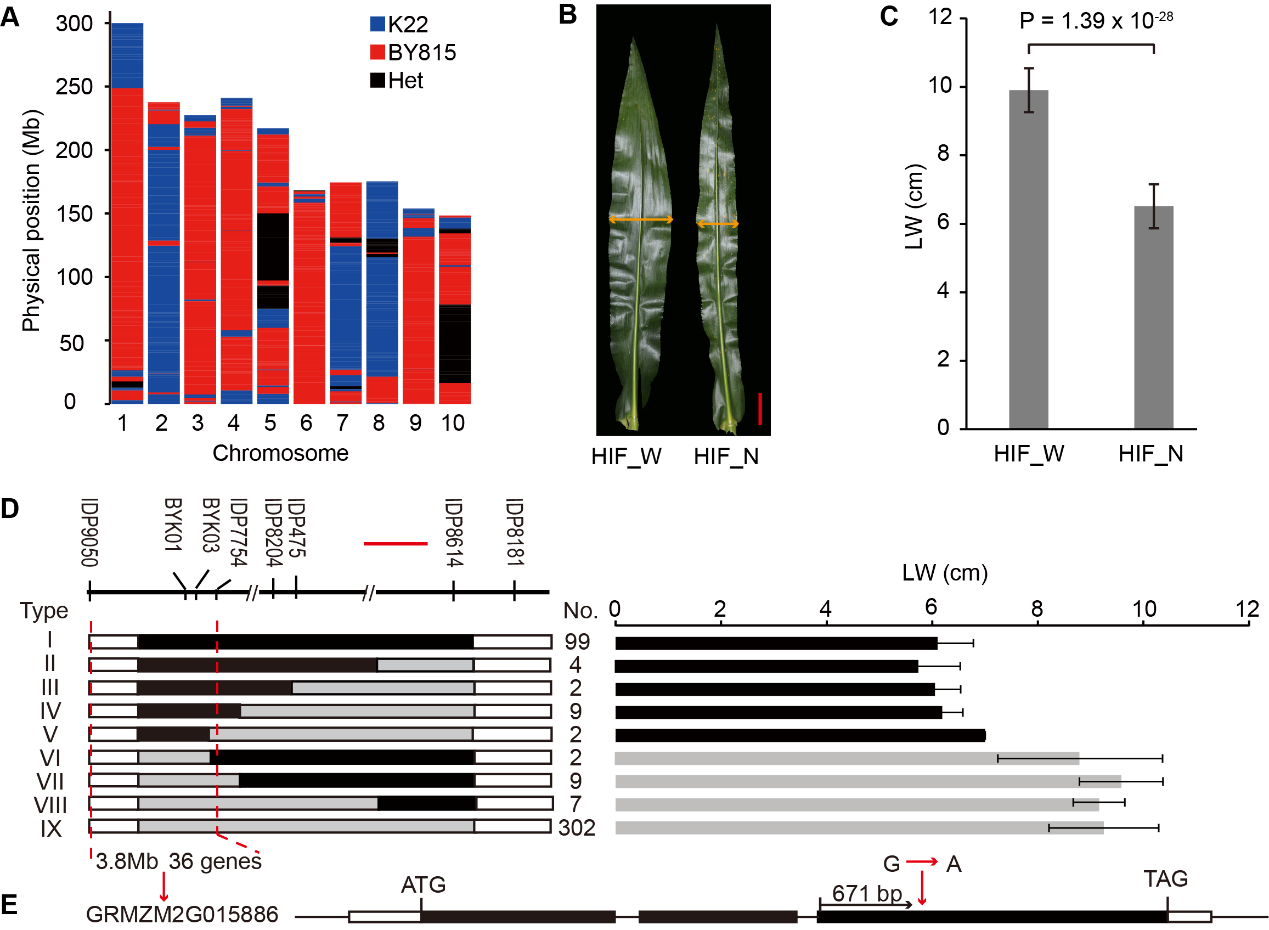
Additional file 4: Figure S1.** Positional cloning of *qLW10* using the HIF family BYK-HIF in the By815 × K22 recombinant inbred line population. (A) Genomic composition of BYK-HIF estimated by Illumina MaizeSNP50K Beadchip. Blue, red, and black regions correspond to K22, By815, and heterozygous regions, respectively. (B) Mature ear leaf of the parents, HIF-W and HIF-N. Scale bar = 5 cm. (C) Statistical analysis of LW between HIF-W and HIF-N. Data are shown as the mean ± SD (n = 30). Student’s t-test was used for analysis. (D) *qLW10* was mapped to a 3.8-Mb genomic DNA region between markers IDP9050 and IDP7754 using 720 F_2_ plants. The numbers (Num) and LW are shown for recombinant plants (II–VIII) and the non-recombinant plants (I and IX). Filled and open bars represent the homozygous HIF-N and heterozygous alleles, respectively. Scale bar = 2 Mb. (E) Allelic variation in the candidate gene GRMZM2G015886 (*ZmCSLD1*) between HIF-W and HIF-N.
