# Supplementary material for: Cellulose synthase-like D1 controls organ size in maize
Source: BMC Plant Biol. 2018 Oct 16;18:239. doi: 10.1186/s12870-018-1453-8 (PMC6192064; doi:10.1186/s12870-018-1453-8)
Supplement: Supplementary file 5 — Figure S2. Characteristics of plant architecture in qLW10MTL and qlw10MTL. (DOCX 683 kb) [file 12870_2018_1453_MOESM5_ESM.docx]

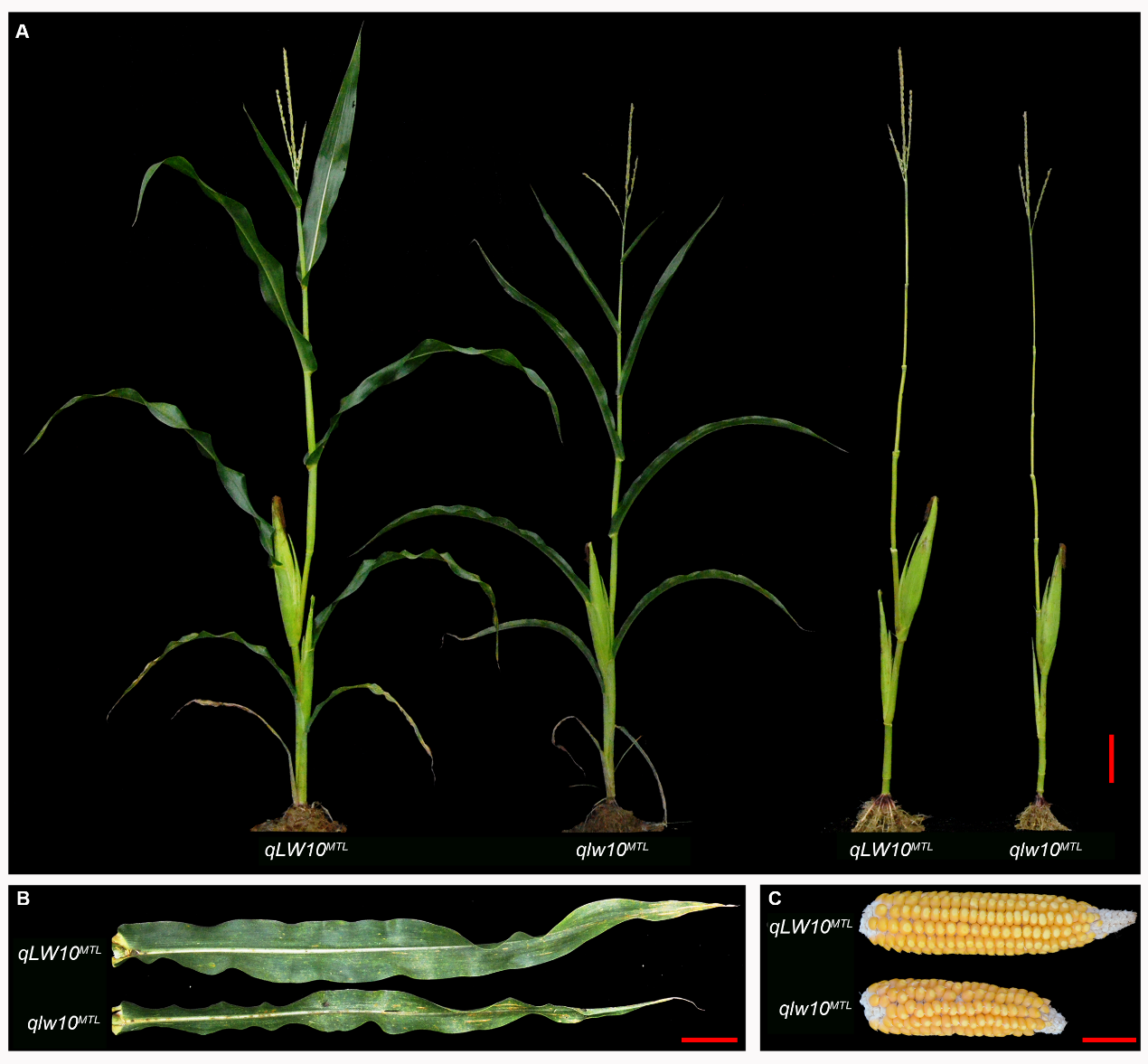


**Additional file 5: Figure S2.** Characteristics of plant architecture in *qLW10^MTL^* and *qlw10^MTL^*. The overall plant (A), leaf (B) and ear (C) characteristics. Scale bar = 10 cm in (A), 5 cm in (B) and 2 cm in (C).
