# Supplementary material for: Cellulose synthase-like D1 controls organ size in maize
Source: BMC Plant Biol. 2018 Oct 16;18:239. doi: 10.1186/s12870-018-1453-8 (PMC6192064; doi:10.1186/s12870-018-1453-8)
Supplement: Supplementary file 7 — Figure S4. Schematic representation of the ZmCSLD1 protein. (DOCX 498 kb) [file 12870_2018_1453_MOESM7_ESM.docx]

**
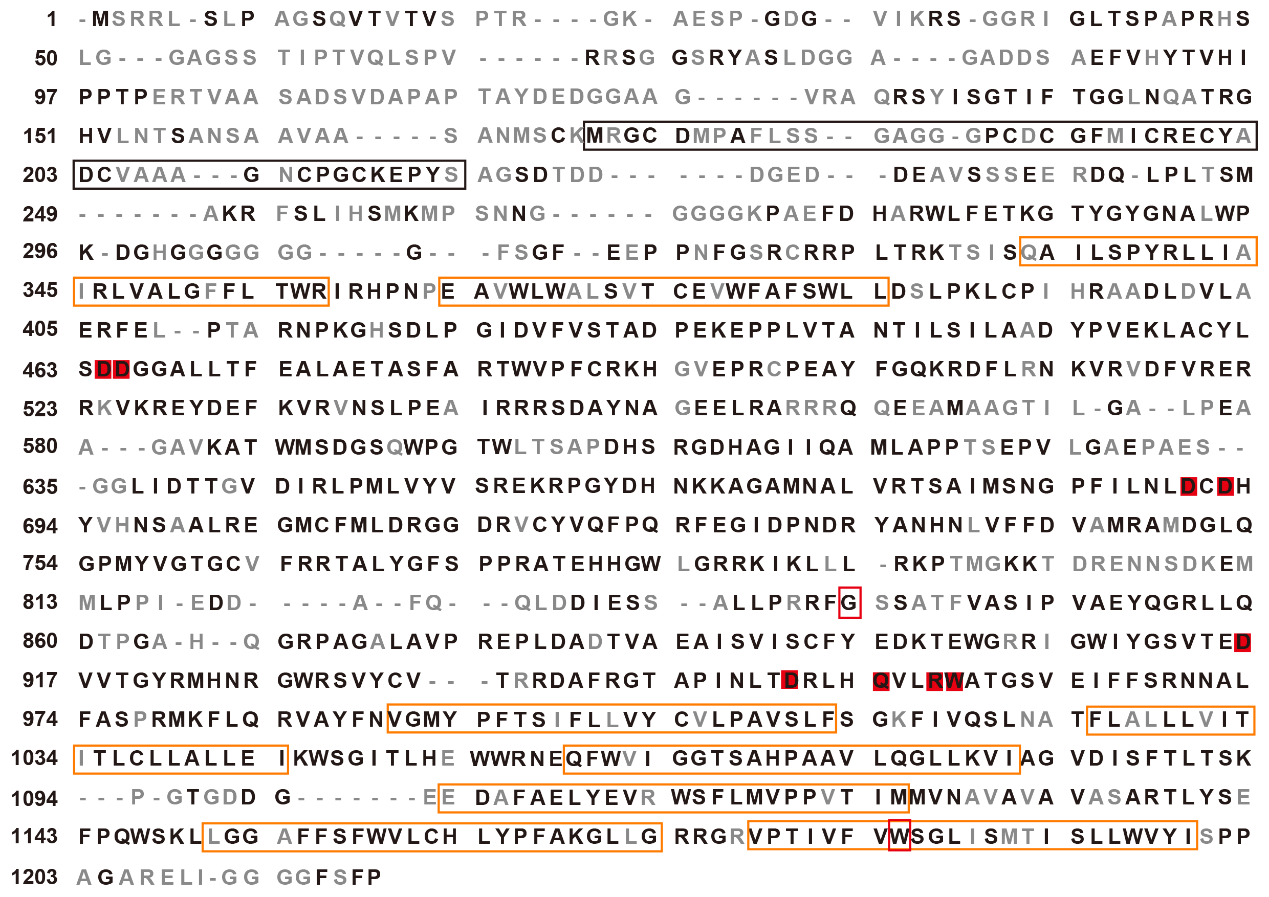
**

**Additional file 7: Figure S4.** Schematic representation of the ZmCSLD1 protein. Black and grey amino acids represent the conserved and variable amino acids, respectively, in the alignment of *ZmCSLD1* orthologs among 32 angiosperms (K7TNG3_MAIZE, C5YPM3_SORBI, K3Z393_SETIT, M0V2Z3_HORVD, I1IHM3_BRADI, CSLD4_ORYSJ, S8E7K7_9LAMI, A0A061DS29_THECC, B9SAX4_RICCO, A0A0B2QGN1_GLYSO, F6HZ78_VITVI, W9RWF3_9ROSA, M1D4L5_SOLTU, A0A067JZN2_JATCU, A0A0D9Y081_9ORYZ, A0A067HGB4_CITSI, V4KZ86_EUTSA, B9GSE5_POPTR, CSLD5_ARATH, A0A0J8ES11_BETVU, R0ILR9_9BRAS, G7KSQ6_MEDTR, V7D0X9_PHAVU, A0A087HKH4_ARAAL, A0A0K9QH13_SPIOL, A0A0L9U9Q0_PHAAN, A0A0S3S0G6_PHAAN, A0A0K9PDM9_ZOSMR, A0A068VET9_COFCA, A0A022S1X9_ERYGU, A0A0B0MYB0_GOSAR, M4EX09_BRARP). Each dash indicates a unique amino acid in other species. The “D,D,D,QXXRW” motif is highlighted in red background. The zinc-finger-like and transmembrane domains are highlighted in black and orange frame, respectively. Two conserved amino acid G839 (mutation in *qlw10^BYK^*) and W1184 (mutation in *Zmcsld1^Mo17^*) were indicated by red frames.
