# Supplementary material for: Cellulose synthase-like D1 controls organ size in maize
Source: BMC Plant Biol. 2018 Oct 16;18:239. doi: 10.1186/s12870-018-1453-8 (PMC6192064; doi:10.1186/s12870-018-1453-8)
Supplement: Supplementary file 8 — Table S4. Genetic design of the allelism tests. (DOCX 14 kb) [file 12870_2018_1453_MOESM8_ESM.docx]

**Additional file 8: Table S4.** Genetic design of the allelism tests

|  | Zmcsld1^W22^ | qLW10^BYK^ | qlw10^BYK^ | qlw10^MTL^ |
| --- | --- | --- | --- | --- |
| qLW10^MTL^ | F_1_ |  |  |  |
| qlw10^MTL^ | F_1_ & F_2_ |  |  |  |
| qLW10^BYK^ | F_1_ |  |  |  |
| qlw10^BYK^ | F_1_ & F_2_ |  |  | F_1_ & F_2_ |
| ZmCSLD1^Mo17^ | F_1_ |  |  |  |
| Zmcsld1^Mo17^ | F_1_ & F_2_ | F_1_ | F_1_ & F_2_ | F_1_ & F_2_ |
