# Supplementary material for: Cellulose synthase-like D1 controls organ size in maize
Source: BMC Plant Biol. 2018 Oct 16;18:239. doi: 10.1186/s12870-018-1453-8 (PMC6192064; doi:10.1186/s12870-018-1453-8)
Supplement: Supplementary file 9 — Figure S5. Box plots showing the four Zmcsld1 allelic effects in six F2 populations. (DOCX 358 kb) [file 12870_2018_1453_MOESM9_ESM.docx]

**
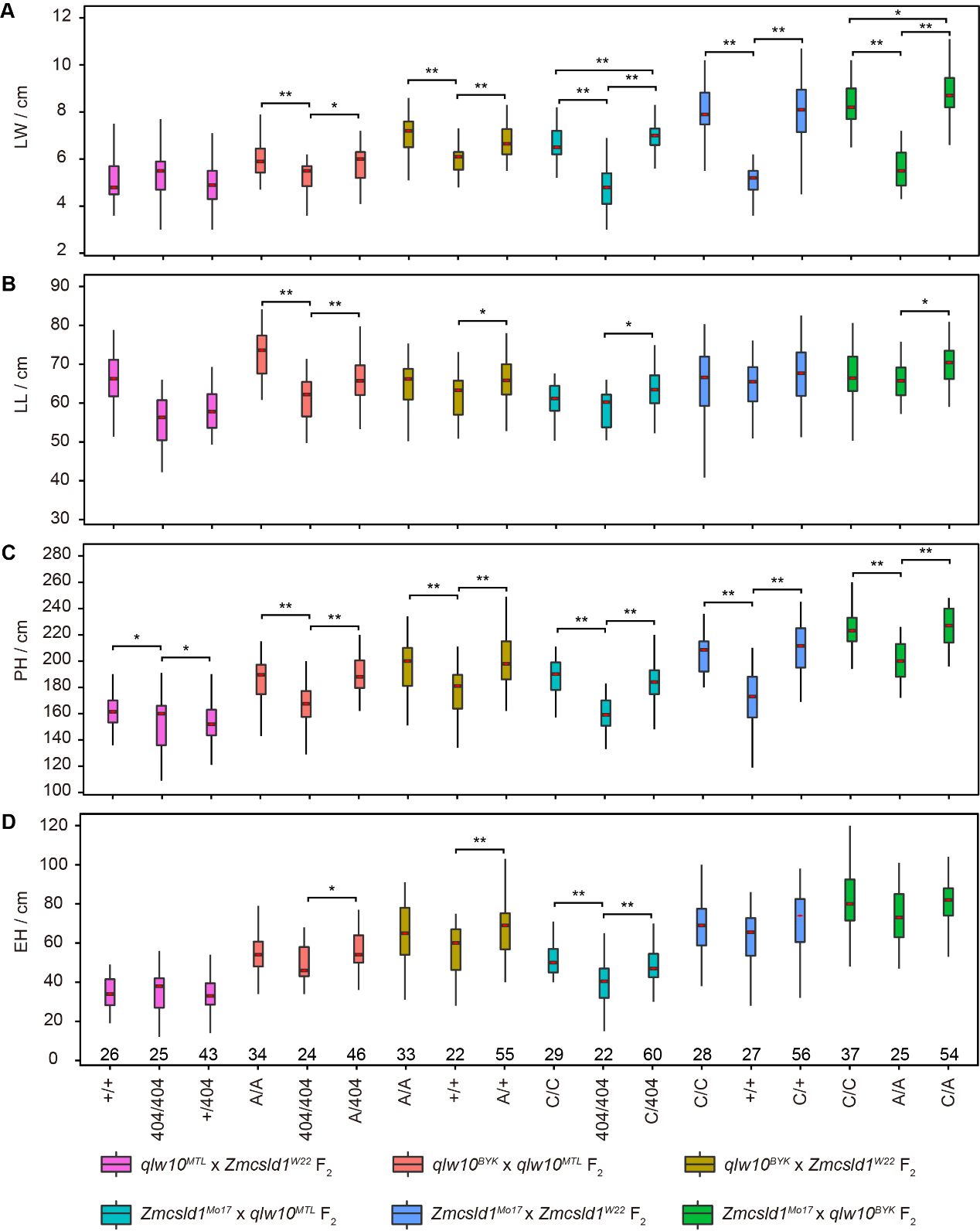
**

**Additional file 9: Figure S5.** Box plots showing the four *Zmcsld1* allelic effects in six F_2_ populations. LW (A), LL (B), PH (C), and EH (D) variations affected by different alleles. + represents the Mu transposon insertion in the *Zmcsld1^w22^* allele, 404 represents the *qlw10^MT^*^L^ allele, A represents the *qlw10^BYK^* allele, and C represents the *Zmcsld1^Mo17^* allele. *P < 0.05, **P < 0.01 (Student’s t-test). The sample size of each allele is shown above the allele types.
