# Supplementary material for: Cellulose synthase-like D1 controls organ size in maize
Source: BMC Plant Biol. 2018 Oct 16;18:239. doi: 10.1186/s12870-018-1453-8 (PMC6192064; doi:10.1186/s12870-018-1453-8)
Supplement: Supplementary file 10 — Figure S6. Phylogenetic tree of CSLD orthologs across 32 angiosperms. (DOCX 256 kb) [file 12870_2018_1453_MOESM10_ESM.docx]

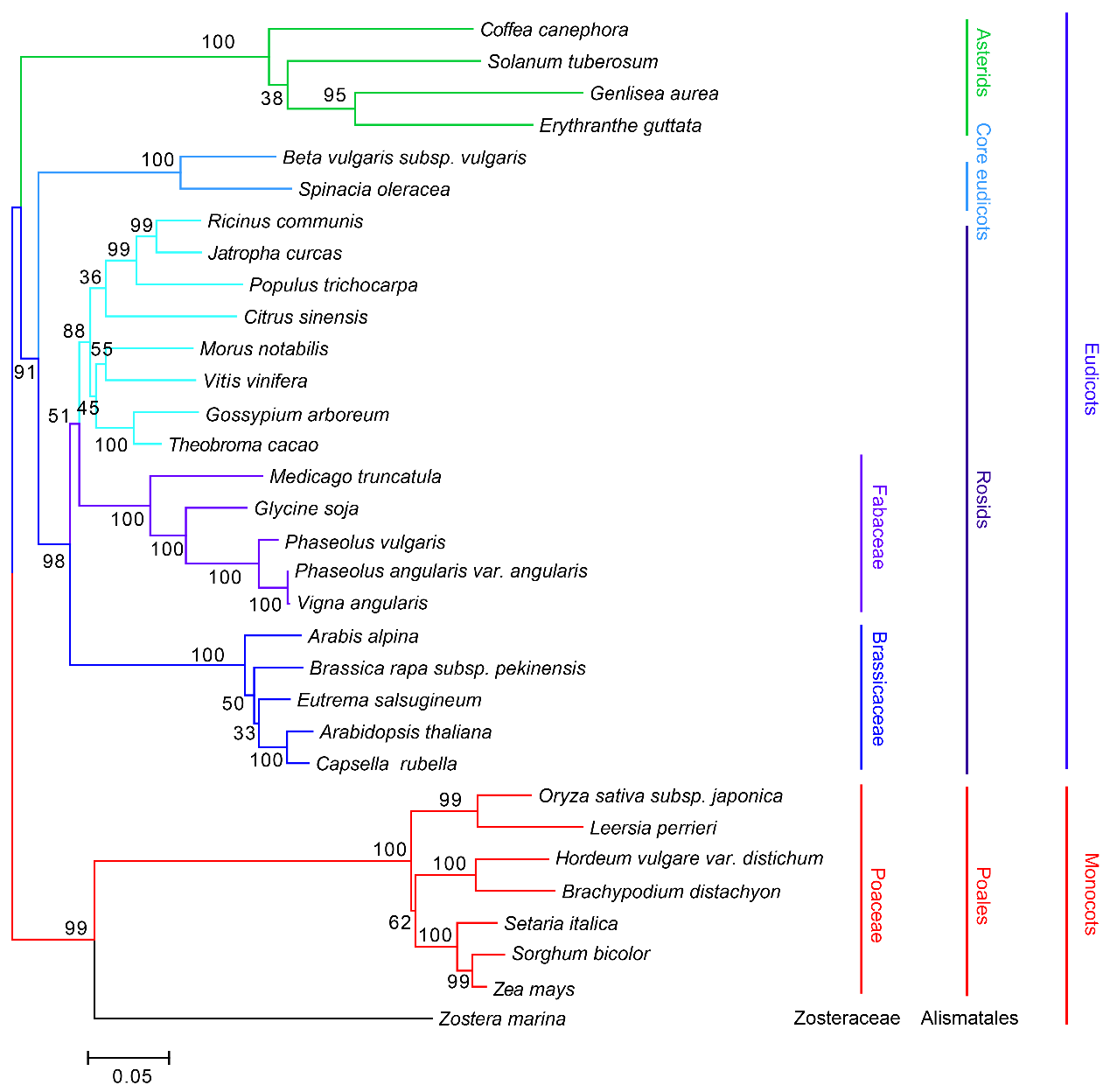


**Additional file 10: Figure S6.** Phylogenetic tree of CSLD orthologs across 32 angiosperms. The tree was constructed on the basis of the whole-protein sequence by the maximum likelihood method with 1000 bootstrap replications.
