# Supplementary material for: Cellulose synthase-like D1 controls organ size in maize
Source: BMC Plant Biol. 2018 Oct 16;18:239. doi: 10.1186/s12870-018-1453-8 (PMC6192064; doi:10.1186/s12870-018-1453-8)
Supplement: Supplementary file 11 — Table S5. Comparisons of cell wall monosaccharide composition and cellulose of leaf blades without leaf veins. (DOCX 16 kb) [file 12870_2018_1453_MOESM11_ESM.docx]

| Sample | Rha | Fuc | Ara | Xyl | Man | Gal | Glc | Cellulose |
| --- | --- | --- | --- | --- | --- | --- | --- | --- |
| *ZmCSLD1^W22^* | 2.50±0.076 | 1.05±0.020 | 32.86±1.92 | 197.82±10.05 | 1.54±0.02 | 13.61±1.05 | 17.67±0.26 | 257.21±7.06 |
| *Zmcsld1^W22^* | 2.43±0.017 | 1.03±0.014 | 34.11±0.60 | 194.79±4.57 | 1.65±0.02** | 12.39±0.13 | 19.32±0.57* | 247.97±7.55 |

**Additional file 11: Table S5.** Comparisons of cell wall monosaccharide composition and cellulose of leaf blades without leaf veins

Alcohol-insoluble residues (AIR) were prepared from leaf blade at ear which veins was excised as described in Method. The results are given as means ± SD (mg/g of AIR) of three biological replicates and five technical replicates per biological replicate. Significance between *ZmCSLD1^W22^* and *Zmcsld1^W22^* was indicated: ** P < 0.01, * P < 0.05 (Student’s t-test).
