# Supplementary material for: Cellulose synthase-like D1 controls organ size in maize
Source: BMC Plant Biol. 2018 Oct 16;18:239. doi: 10.1186/s12870-018-1453-8 (PMC6192064; doi:10.1186/s12870-018-1453-8)
Supplement: Supplementary file 12 — Figure S7. The pleiotropic effects of Zmcsld1 estimated by comparing Zmcsld1 and ZmCSLD1 homologous lines among different alleles. (DOCX 114 kb) [file 12870_2018_1453_MOESM12_ESM.docx]

**
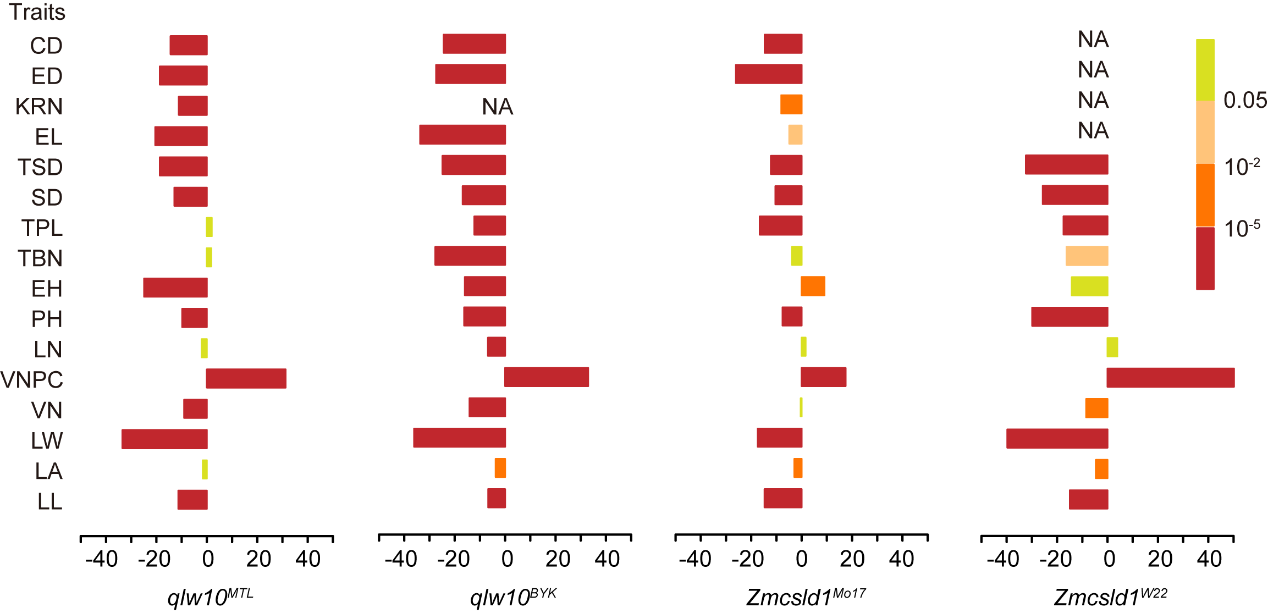
**

**Additional file 12: Figure S7.** The pleiotropic effects of *Zmcsld1* by comparing *Zmcsld1* and *ZmCSLD1* homologous lines among different alleles. The significance levels of the differences analysed by Student’s t-test (n = 30) are indicated in different colours. Traits abbreviations are listed in Additional file 2: Table S2.
