# Supplementary material for: Cellulose synthase-like D1 controls organ size in maize
Source: BMC Plant Biol. 2018 Oct 16;18:239. doi: 10.1186/s12870-018-1453-8 (PMC6192064; doi:10.1186/s12870-018-1453-8)
Supplement: Supplementary file 13 — Figure S8. Epidermal impressions of the fourth leaf abaxial surfaces from 40-day-old seedlings. (DOCX 1468 kb) [file 12870_2018_1453_MOESM13_ESM.docx]

**
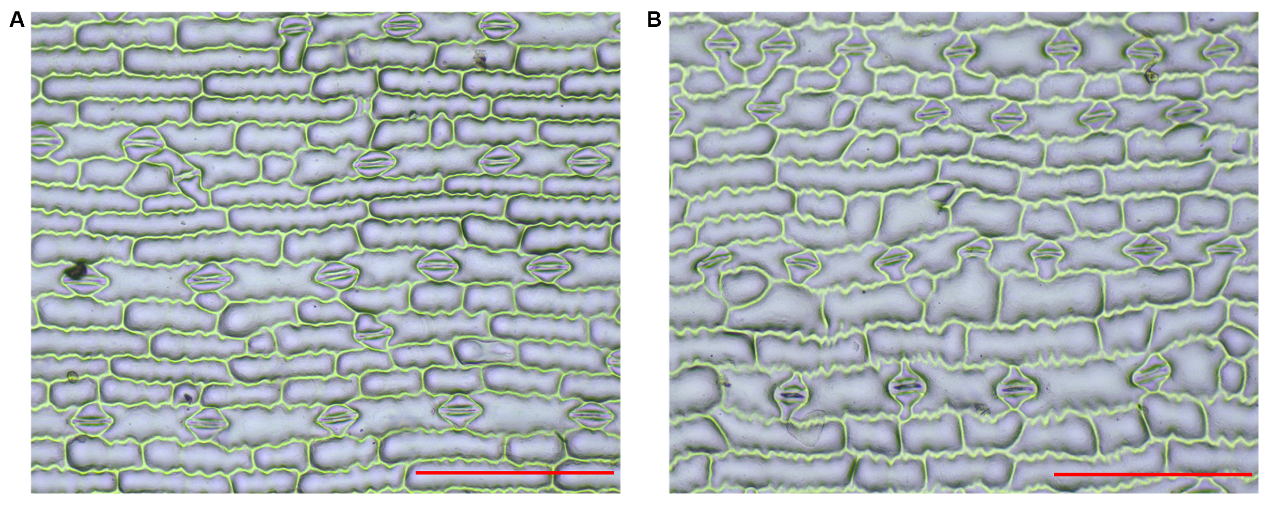
Additional file 13: Figure S8.** Epidermal impressions of the fourth leaf abaxial surfaces from 40-day-old seedlings. *qLW10^MTL^* (A) and *qlw10^MTL^* (B) were observed under a light microscope. Scale bar = 200 μm.
