# Supplementary material for: Cellulose synthase-like D1 controls organ size in maize
Source: BMC Plant Biol. 2018 Oct 16;18:239. doi: 10.1186/s12870-018-1453-8 (PMC6192064; doi:10.1186/s12870-018-1453-8)
Supplement: Supplementary file 14 — Figure S9. Comparisons of leaf and cell size between ZmCSLD1 and Zmcsld1 in other three alleles. (DOCX 311 kb) [file 12870_2018_1453_MOESM14_ESM.docx]

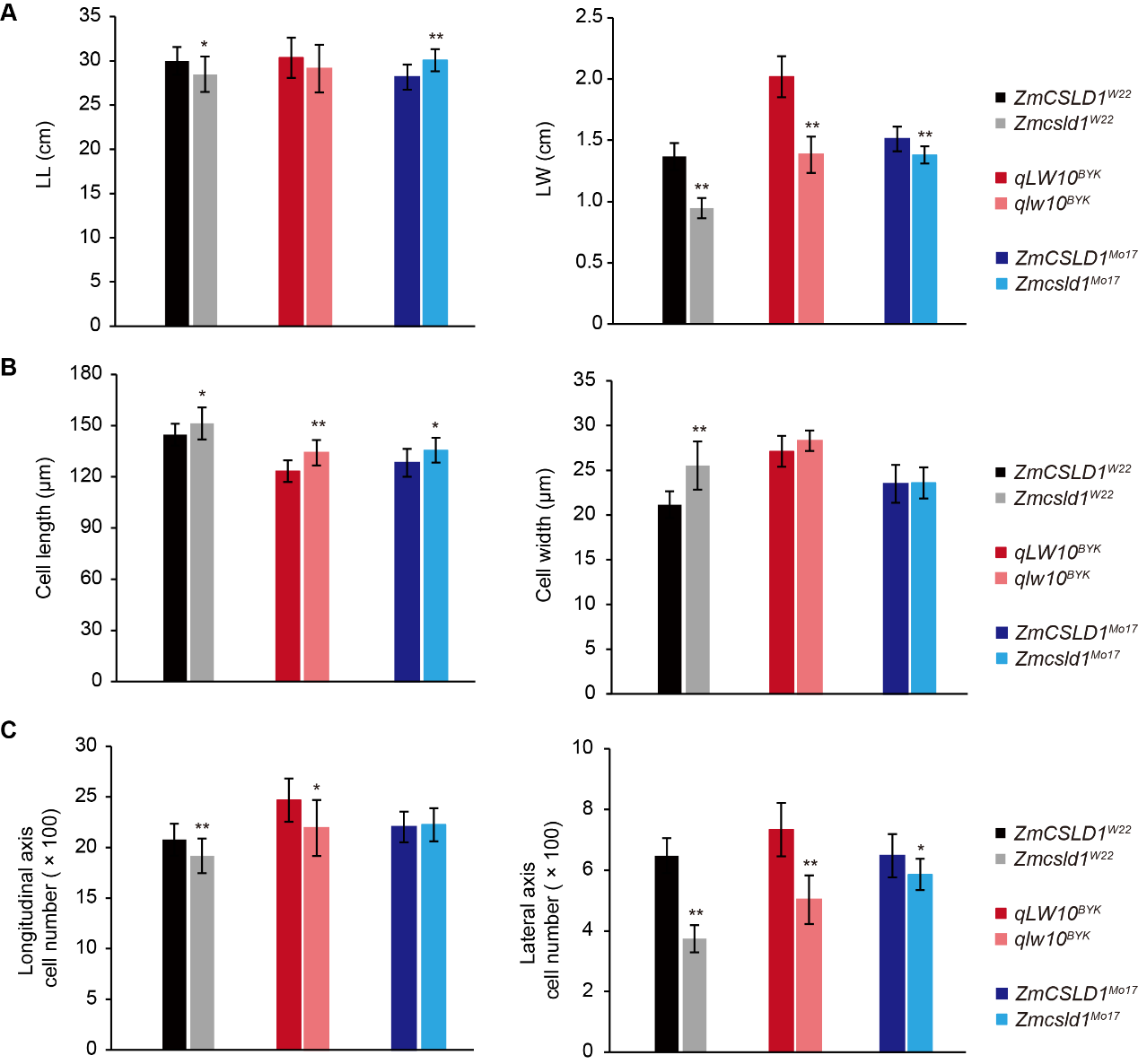


**Additional file 14: Figure S9.** Comparisons of leaf and cell size between *ZmCSLD1* and *Zmcsld1* in other three alleles. (A) LL and LW of the third unfolded leaf from 20-day-old seedlings. (B) Cell length and width of the third leaf abaxial epidermis cell. Fifty noraml cells per plant were measured by ImageJ for the average cell width and length. (C) The number of cells in the longitudinal and lateral axis of the leaf blades was estimated by dividing the average LL by the cell length and the average LW by the cell width, respectively. The data are shown as the mean ± SD (n = 10 to 20). ** P < 0.01, * P < 0.05 (Student’s t-test).
