# Supplementary material for: Cellulose synthase-like D1 controls organ size in maize
Source: BMC Plant Biol. 2018 Oct 16;18:239. doi: 10.1186/s12870-018-1453-8 (PMC6192064; doi:10.1186/s12870-018-1453-8)
Supplement: Supplementary file 15 — Figure S10. Expression pattern of ZmCSLD1 in various tissues at different developmental stages. (DOCX 238 kb) [file 12870_2018_1453_MOESM15_ESM.docx]

**
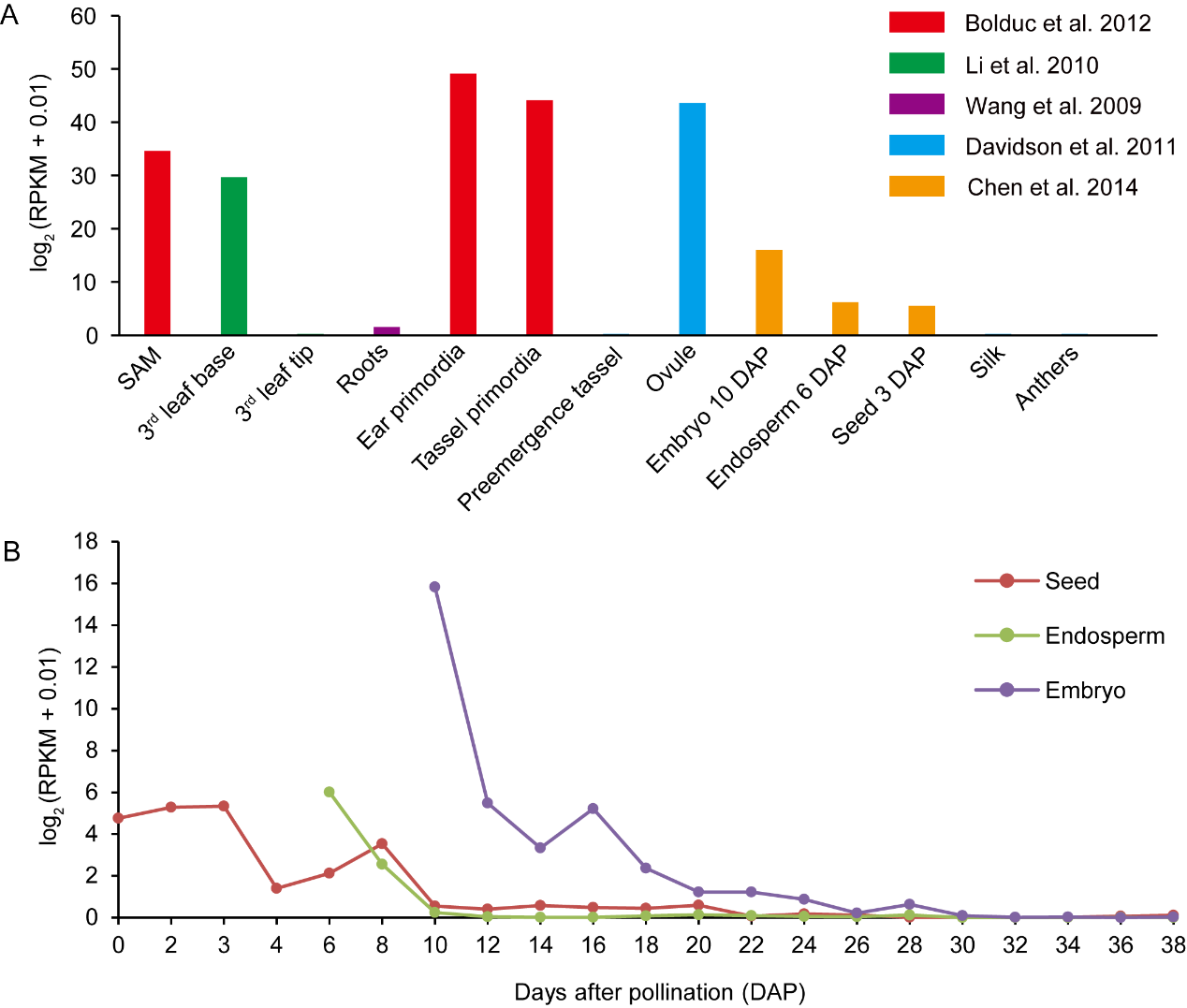
Additional file 15: Figure S10.** Expression pattern of *ZmCSLD1* in various tissues at different developmental stages. (A) *ZmCSLD1* expression in 13 tissues according to five studies shown in different colors. (B) Sequential expression of *ZmCSLD1* in 15 embryo (from 10 DAP to 38 DAP per 2 DAP), 17 endosperm (from 6 DAP to 38 DAP per 2 DAP), and 21 whole seed (from 0 DAP to 38 DAP per 2 DAP) samples of B73 materials. Expression values were normalized by log_2_ (RPKM+0.01) cited from Chen et al.’s expression data [40].
