# Supplementary material for: Cellulose synthase-like D1 controls organ size in maize
Source: BMC Plant Biol. 2018 Oct 16;18:239. doi: 10.1186/s12870-018-1453-8 (PMC6192064; doi:10.1186/s12870-018-1453-8)
Supplement: Supplementary file 16 — Figure S11. Relative expression of histone H2B and CycD3;1b in root, shoot and leaf tissues in qLW10MTL and qlw10MTL. (DOCX 166 kb) [file 12870_2018_1453_MOESM16_ESM.docx]

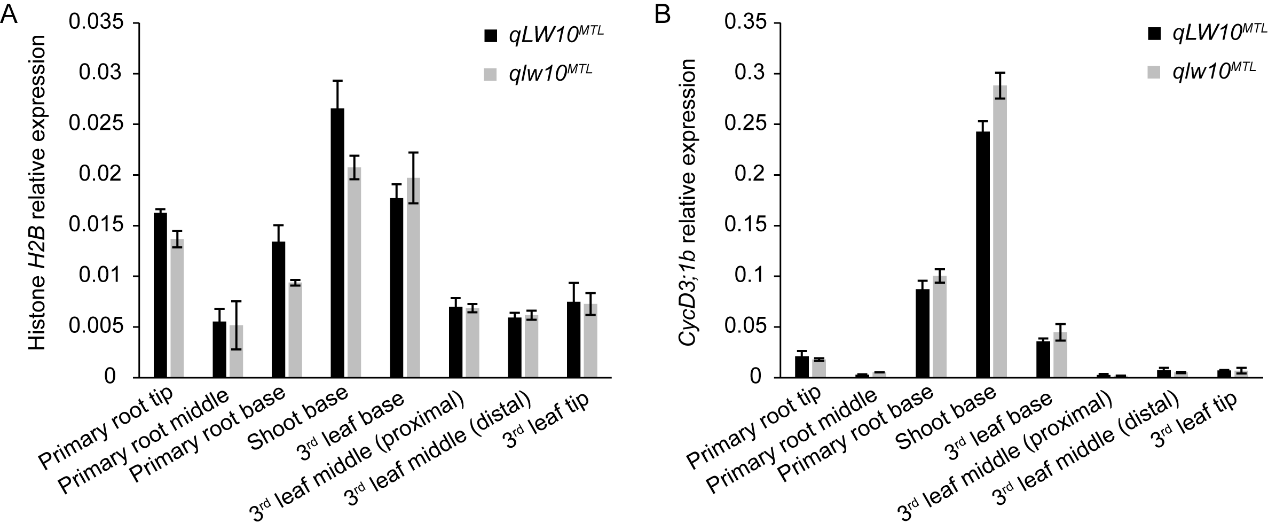


**Additional file 16: Figure S11.** Relative expression of histone *H2B* (A) and *CycD3;1b* (B) in eight tissues corresponding to **Fig.6a** and **6b** in *qLW10^MTL^* and *qlw10^MTL^* were detected by qRT-PCR, data are shown as the mean ± SE (n=3).
