# Supplementary material for: Cellulose synthase-like D1 controls organ size in maize
Source: BMC Plant Biol. 2018 Oct 16;18:239. doi: 10.1186/s12870-018-1453-8 (PMC6192064; doi:10.1186/s12870-018-1453-8)
Supplement: Supplementary file 17 — Table S6. List of primers used in this study. (DOCX 18 kb) [file 12870_2018_1453_MOESM17_ESM.docx]

**Additional file 17: Table S6.** List of primers used in this study

|  | Name | Forward | Reverse |
| --- | --- | --- | --- |
| *ZmCSLD1* sequencing | CSLD1-P1 | 5’-TTTGGCCCGTGAATTAGG-3’ | 5’-GACCAACCAGCGAAACTA-3’ |
|  | CSLD1-P2 | 5’-TAGTTTCGCTGGTTGGTCG-3’ | 5’-CTGGGCATCTTCATGGAGTGT-3’ |
|  | CSLD1-P3 | 5’-TGCAAGGAGCCCTACTCTG-3’ | 5’-GCCTTCTTGTTGTGGTCGT-3’ |
|  | CSLD1-P4 | 5’-TTCTTGGAGCGTTGCCTGAG-3’ | 5’-CCGATCACCCAGAACTGC-3’ |
|  | CSLD1-P5 | 5’-CGTCATCACCATTACGCTCT-3’ | 5’-GCCACTTGGACTGGTAACGA-3’ |
| Fine  mapping | IDP8182 | 5’-TGGACGACTACTCGGTCTCC-3’ | 5’-GGCATGGAGTTGATCACAGC-3’ |
|  | IDP9050 | 5’-GTACGAGAAGGACCTGAGCG-3’ | 5’-CTCTCGCTGTGCATTGACC-3’ |
|  | IDP4274 | 5’-CTAGCAGGAGAAGTAAAC-3’ | 5’-TCAGTCAACGCCTAAAGA-3’ |
|  | MTL02 | 5’-CCGTGTTCCAAGACAAGGTT-3’ | 5’-AGCTGGCACCGATGATTAG-3’ |
|  | IDP7754 | 5’-TACATGGCATGAGATGGAGG-3’ | 5’-ATCTGAGAGGCCGATTTGG-3’ |
|  | BYK01 | 5’-AGGTGAGCAGTTGGGATG-3’ | 5’-GTGTGCATTATTTTCTCCCTAC-3’ |
|  | BYK03 | 5’-CGTATTTGTCTGTCGTCC-3’ | 5’-ACCGTTTTTGTGTAAGGCG-3’ |
|  | IDP8204 | 5’-TCTTTCAGTGGTACCCGTCC-3’ | 5’-ACTCTTGCGGCAGTAGTTGC-3’ |
|  | IDP475 | 5’-AGTCACATGTTGATACGCGG-3’ | 5’-AGTCACCCACCTTTCTTTGG-3’ |
|  | IDP8614 | 5’-GTATCTAGCGAGCGCATTCC-3’ | 5’-TGCTATTCCTGAATACCGGG-3’ |
|  | IDP8181 | 5’-AGAATTGTCGTGGCAGAAGC-3’ | 5’-GATTCAAGAACCGAGAAGGC-3’ |
| GFP vector construction | rCSLD1-03^a^ | 5’-AGT**CTCGAG**TTTCTGGGAGCGATGTC-3’ | 5’-GAC**ACTAGT**CAACGGGAAGCTGAATCCG-3’ |
| Allelism test | Mo17-T/C^b^ | 5’-GTGTGCCGACCATCGTGTTCGT-3’ | FAM tail + 5’-TGGAGATGAGCCCCGACCG-3’ &  VIC tail + 5’-ATGGAGATGAGCCCCGACCA-3’ |
|  | BYK-G/A^b^ | FAM tail + 5’-CTCCCGCGGCGGTTCGG-3’ &  VIC tail + 5’-GCTCCCGCGGCGGTTCGA-3’ | 5’-GGATGGACGCCACGAACGT-3’ |
|  | TIR6 | 5’-AGAGAAGCCAACGCCAWCGCCTCYATTTCGTC-3’ | |
| qRT-PCR | qCSLD1-04 | 5’-GAAGACCAGCATATCTCAGG-3’ | 5’-GAAAGCGCCCACAACCAC-3’ |
| qRT-PCR | qCcyB1;4 | 5’-TCGTCCCTCCCGCATTCTCT-3’ | 5’-GTGAGGGAAGTTGCAGCTAG-3’ |
|  | qCycD1;3 | 5’-CGAAGGCTACTTTATCCGTG-3’ | 5’-CAACCGACTCTGAGAACAGC-3’ |
|  | qH2B | 5’-CCAGCAGCCTTCTTTCGCA-3’ | 5’-GGTTTCGGGATTGGGCTTC-3’ |
|  | TUBG1 | 5’-AATGGGATTCAGAACAGCG-3’ | 5’-CAGCAATAGAGTGGCACAG-3’ |
| Yeast two-hybrid | AD-GS1^c^ | 5’-CCATGGAGGCCAGTGAATTC  TTTCTGGGAGCGATGTCG-3’ | 5’-CAGCTCGAGCTCGATGGATC  ATATGCTGGTCTTCCTGG-3’ |
|  | AD-GS2^c^ | 5’-GCCATGGAGGCCAGTGAATTC  CTGGATGTCCTTGCCGAG-3’ | 5’-CAGCTCGAGCTCGATGGATCA  GCGCTGGAGGAACTTCATC-3’ |
|  | BD-GS1^c^ | 5’-ATGGCCATGGAGGCCGAATTC  TTTCTGGGAGCGATGTCG-3’ | 5’-CCGCTGCAGGTCGACGGATC  ATATGCTGGTCTTCCTGG-3’ |
|  | BD-GS2^c^ | 5’-ATGGCCATGGAGGCCGAATTC  CTGGATGTCCTTGCCGAG-3’ | 5’-CCGCTGCAGGTCGACGGATCA  GCGCTGGAGGAACTTCATC-3’ |

^a^ Bold text are the additional restriction site CTCGAG for XhoI and ACTAGT for SpeI

^b^ FAM tail sequence: 5’-GAAGGTGACCAAGTTCATGCT-3’; VIC tail sequence: 5’-GAAGGTCGGAGTCAACGGATT-3’.

^c^ Underline text are the vector sequence used for overlapping.
